# Supplementary material for: Identification of Freezing-Responsive microRNAs and Their Targets in Chinese Jujube by Small RNA and Degradome Sequencing
Source: Int J Mol Sci. 2026 Sep 4;27(17):7912. doi: 10.3390/ijms27177912 (PMC13566890; doi:10.3390/ijms27177912)
Supplement: Supplementary file 1 [file ijms-27-07912-s001.zip › Supplementary Material S2/novel_miR_188.pdf]

Provisional ID	:	novel_miR_188
Score total	:	0.6
Score for star read(s)	:	-1.3
Score for read counts	:	0
Score for mfe	:	0.3
Score for randfold	:	1.6
Score for cons. seed	:	
Total read count	:	140253
Mature read count	:	140253
Loop read count	:	0
Star read count	:	0

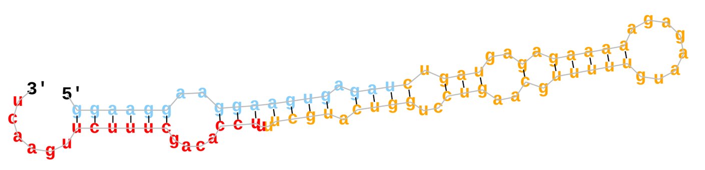

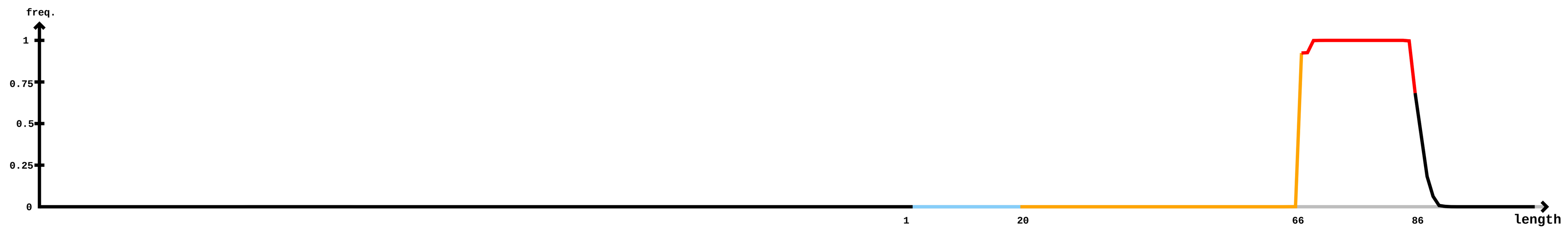

Star Mature

5'- ucucuuucucucucucucucucuaaaaaaacaaaaggaaagaaaaaaaaaaaaaaaaacuuacuacuacuacuaguaguauaucuucuaauuugcuggaaaggcacaccaaagagaaagaaauuaaaccaaggaaggaaggaaggaaggaaggaagugagaucugaugagagaaaaagagaauguuuuugcaaguccuggucaugcuuuuccacagcuuucuugaacuuucuuucuuucuguuuagucu -3' exp

..((((((((.....(((.(((...........))).)))..................((((((((....))))))))...........((((.....)))).....)))))))))).((((((..((((((((((((((..(((((((..((((((.(((((.((.((....((((....)))).....)))).)))).)..))).)))..)))))))...))))))))))))))))))))..

read	reads	mm	sample
ucucuuucuGucucucuc	5	1	LT-2
...cuuuGucucucucucucucu	1	1	LT-3
.......cucucucucuUucucuaa	1	1	LT-1
Aaaaaggaaagaaaaaaaaaa	5	1	LCK-3
aaaaggaaagaaaaaaaaaaaaa	1	0	JT-1
aaaaggaaagaaaaaaaaaaaaaaa	1	0	JT-1
aUggaaagaaaaaaaaaaa	1	1	LT-1
aggaaGgaaaaaaaaaaaaaaaaaa	1	1	LT-3
aNaaaaaaaaaaaaaaaaa	1	1	JCK-3
uuuuccacagcuuucuugaac	6	0	JCK-2
uuuuccacagcuuucuugaac	4	0	JCK-1
uuuuccacagcuuucuugaac	7	0	JCK-3
uuuuccacagcuuucuugaac	3	0	LT-1
uuuuccacagcuuucuugaac	3	0	LCK-3
uuuuccacagcuuucuugaac	3	0	JT-3
uuuuccacagcuuucuugaac	2	0	JT-2
uuuuccacagcuuucuugaac	3	0	LT-3
uuuuccacagcuuucuugaacu	1	0	JCK-3
uuuuccacagcuuucuugaacu	4	0	JCK-2
uNuuccacagcuuucuugaacu	1	1	JCK-3
uuuccacagcuuucuuga	2	0	JCK-1
Auuccacagcuuucuugaacu	1	1	JCK-3
uuuccacagcuuucuugaacu	2	0	LCK-3
uuuccacagcuuucuugaacu	1	0	JCK-2
Auuccacagcuuucuugaacu	2	1	JCK-2
Guuccacagcuuucuugaacu	2	1	LT-3
uuuccacagcuuucuugaacu	1	0	LT-1
uuuccacagcuuucuugaacu	1	0	JCK-3
Cuuccacagcuuucuugaacuu	3	1	JCK-2
Cuuccacagcuuucuugaacuu	2	1	JCK-1
Cuuccacagcuuucuugaacuu	3	1	LT-1
Cuuccacagcuuucuugaacuu	3	1	LT-3
Cuuccacagcuuucuugaacuu	1	1	LT-2
Cuuccacagcuuucuugaacuuu	2	1	JCK-1

Star Mature

ucucuuucucucucucucucuaaaaaaacaaaaggaaagaaaaaaaaaaaaaaaaaaacuuacuacuacuacuaguaguauaucuucuaauuugcuggaaaggcacaccaaagagaaagaaauuaaaccaaggaaggaaggaaggaaggaaggaagugagaucugaugagagaaaaagagaauguuuuugcaaguccuggucaugcuuuuccacagcuuucuugaacuuucuuucuuucuguuuagucu

Cuuccacagcuuucuugaacuuu	1	1	LT-1
Cuuccacagcuuucuugaacuuu	3	1	LCK-3
Cuuccacagcuuucuugaacuuu	1	1	LT-3
uuccacagcuuucuugaa	9	0	JCK-3
uuccacagcuuucuugaa	43	0	LT-2
uuccacagcuuucuugUa	2	1	JCK-3
uuccacagcuuucuugaa	15	0	JT-3
uuccacagcuuucuugaC	4	1	LT-2
uuccacagcuuucuugaa	15	0	JCK-2
uuccacagcuuucuugUa	1	1	LT-1
uuccacagcuuucuugCa	1	1	LT-2
uuccacagcuuucuugaU	11	1	LT-1
uuccacagcuuucuugaU	14	1	LT-3
uuccacagcuuucuugaa	53	0	LCK-2
uuccacagcuuucuugaU	1	1	JCK-1
uuccaNagcuuucuugaa	1	1	LCK-1
uuccacagcuuucuugaa	43	0	LCK-3
uuccacagcuuucuugGa	1	1	LCK-2
uuccacagcuuucuugaa	52	0	LT-3
uuccacagcuuucuugUa	19	1	LCK-3
uuccacagcuuucuugaU	1	1	JCK-2
uuccacagcuuucuugUa	4	1	LT-3
uuccacagcuuucuugaU	3	1	LT-2
uuccacagcuuucuuUaa	1	1	LT-1
uuccacagcuuucuugaa	5	0	JT-2
uuccacagcuuucuGgaa	1	1	LCK-1
uuccacagcuuucuugaU	4	1	LCK-3
uuccacagcuuucuugUa	15	1	LT-2
uuccacagcuuucuugaC	1	1	LT-3
uuccacagcuuucuugaC	1	1	JCK-3
uuccacagcuuucuugaa	16	0	JCK-1
uuccacagcuuucuugaa	31	0	LT-1
uuccacagcuuucuugUa	5	1	JT-1
uuccacagcuuucuugaa	46	0	LCK-1
uuccacCgcuuucuugaac	10	1	LT-2
uuccacagcuuucuugaCc	1	1	JT-1
uuccacagcuuucuugGac	1	1	JCK-3
uuccacagcuuuNuugaac	1	1	LCK-1
uuccacagcuuucuGgaac	1	1	JCK-2
uuUcacagcuuucuugaac	6	1	JCK-1
uuccacagcuuucuugaac	8415	0	LCK-3
uuccCcagcuuucuugaac	2	1	JT-1
uuccacagcuuucuugUac	1	1	LT-3
uuccacagcCuucuugaac	5	1	LT-3
uuccacagcuuucuugaUc	2	1	LT-3
uuccacagcuuucuugaac	1676	0	LCK-1
uuccacaAcuuucuugaac	2	1	LCK-3
uuccacaAcuuucuugaac	5	1	LT-1
uuAcacagcuuucuugaac	1	1	LT-3
uNccacagcuuucuugaac	1	1	LCK-2
uuccacagcuuucuugaaU	64	1	LT-2
uCccacagcuuucuugaac	1	1	JCK-2
uuccacagcuuucuugaCc	1	1	JCK-3
uuccacagcuuucuugaCc	7	1	JCK-2
uuccacagcuuucCugaac	1	1	LT-2
uCccacagcuuucuugaac	1	1	LCK-1
uuccacCgcuuucuugaac	11	1	LCK-3
uuccCcagcuuucuugaac	4	1	JCK-2
uuccCcagcuuucuugaac	2	1	JCK-3
uuccacagcuGucuugaac	3	1	JCK-1
uuccacagcuuucuugaGc	1	1	LT-3
uuccCcagcuuucuugaac	13	1	LCK-3
uuccacagcuuucuugaac	5694	0	LT-2
uuccacaUcuuucuugaac	2	1	JCK-2
uuccacagcuuCcuugaac	3	1	JCK-2
uuccacagcuuucuugaac	1739	0	LCK-2
uuccacagcuuucuugaaU	37	1	LT-1
uuccCcagcuuucuugaac	12	1	LT-1
uuccacaAcuuucuugaac	1	1	LT-3
uuccacagcCuucuugaac	1	1	LT-2

Star Mature

ucucuuucucucucucucucucucuaaaaaaacaaaaggaaagaaaaaaaaaaaaaaaaacuuacuacuacuacuaguaguauaucuucuaauuugcuggaaaggcacaccaaagagaaagaaauuaaaccaaggaaggaaggaa ggaaggaaggaagugagau cugaugagagaaaaagagaauguuuuugcaaguccuggucaugcuu uuccacagcuuucuugaacu uucuuucuuucuguuuagucu

uuccacagcuuuUuugaac	2	1	JCK-3
uuccacagcuuucuugCac	5	1	LT-2
uuccacagcuuucuugaac	411	0	JT-2
uuccacagcuuucuugUac	3	1	LCK-3
uuccacagcuuucuugCac	4	1	LT-1
uuccacagcuuuAuugaac	2	1	LT-1
uuccaAagcuuucuugaac	1	1	JCK-2
uuccGcagcuuucuugaac	1	1	LT-1
uNccacagcuuucuugaac	2	1	JCK-3
uuccacagcuuucuugaCc	13	1	LT-2
uCccacagcuuucuugaac	1	1	JCK-1
uuccacagUuuucuugaac	4	1	LCK-3
uuccacagcuuucCugaac	3	1	JCK-2
uuccacagcuuuUuugaac	3	1	LT-1
uuccacagcuuucuugaGc	1	1	JCK-1
uuccacagcuuucuugaCc	10	1	LT-3
uucUacagcuuucuugaac	1	1	JCK-3
uuccacagcuuucuugaac	542	0	JT-3
Cuccacagcuuucuugaac	2	1	JT-1
uuccacagcuuucuugCac	1	1	JCK-3
uuccacagcuCucuugaac	1	1	LT-1
uuccacagcuGucuugaac	1	1	LT-1
uuccacaAcuuucuugaac	2	1	LT-2
uuccacagcuuucuugaCc	8	1	LCK-3
uuccacagcuuCcuugaac	2	1	JCK-3
Cuccacagcuuucuugaac	3	1	LCK-3
Cuccacagcuuucuugaac	4	1	LT-1
uuccacaAcuuucuugaac	1	1	JCK-2
uuccacGgcuuucuugaac	2	1	JCK-2
uuccacagcuuucuugaUc	10	1	LCK-3
uuccacCgcuuucuugaac	12	1	LT-1
uuccacagcCuucuugaac	7	1	LT-1
uuccacagcuuucuugaaU	103	1	LCK-3
uuccaNagcuuucuugaac	3	1	LCK-1
uuccacagcuuucuuUaac	1	1	JT-1
uuccacagcuuucCugaac	1	1	JCK-3
uuccacagcuuucuugaaU	53	1	JCK-2
uuccacagcuuuUuugaac	1	1	LCK-2
uuccacagcuuucuugCac	2	1	JCK-1
uuccacCgcuuucuugaac	2	1	LCK-2
uuccacagcuuCcuugaac	3	1	JCK-1
uuccacagcuuucuugaaU	24	1	JCK-1
uuccacagcuuucuugaaU	98	1	LT-3
uuUcacagcuuucuugaac	1	1	JCK-3
uuGcacagcuuucuugaac	3	1	LT-2
uuccacagcuuucuugaac	1423	0	JT-1
uuccacagUuuucuugaac	1	1	LT-3
uuccacCgcuuucuugaac	1	1	JT-2
uuccacagcuuucuugaUc	3	1	JT-1
Cuccacagcuuucuugaac	1	1	JCK-3
uuccacagcuuucuugaac	3571	0	JCK-2
uuUcacagcuuucuugaac	2	1	JCK-2
uuccaUagcuuucuugaac	2	1	LT-3
uuccacagcuuucuugaac	2180	0	JCK-1
uuccacagcuuCcuugaac	2	1	LT-1
uuccCcagcuuucuugaac	1	1	JCK-1
uuccacagcuuucuugaUc	2	1	JCK-1
uuccacagcCuucuugaac	1	1	JCK-2
uuccaUagcuuucuugaac	1	1	LT-2
uuccacagcuuucCugaac	2	1	LCK-3
uuccacaAcuuucuugaac	1	1	JCK-3
uuccCcagcuuucuugaac	33	1	LT-2
uuccacagcuuucuuAaac	1	1	JCK-1
uuccacagcCuucuugaac	1	1	JCK-1
uuccacagcuuucuCgaac	1	1	LT-1
uuccacagcuuuAuugaac	1	1	LT-2
uuccacagcuuucuugaaU	28	1	JCK-3
uuccacagcuuuUuugaac	8	1	JCK-2
uuccaAagcuuucuugaac	1	1	LT-2
uuccacagcuuuUuugaac	6	1	LT-2

```
Star                                                                                          Mature
ucucuuucucucucucucucuaaaaaaacaaaaggaaagaaaaaaaaaaaaaaaaaaacuuacuacuacuacuaguaguauaucuucuaauuugcuggaaaggcacaccaaagagaaagaaauuaaaccaaggaaggaaggaaggaaggaaggaagugagaucugaugagagaaaaagagaauguuuuugcaaguccuggucaugcuuuuccacagcuuucuugaacuuucuuucuuucuguuuagucu
.......................................................................................................................................................................................................................uuccacagAuuucuugaac......................   1   1   LT-2
.......................................................................................................................................................................................................................uucNacagcuuucuugaac......................   1   1   JCK-1
.......................................................................................................................................................................................................................uuAcacagcuuucuugaac......................   1   1   JCK-1
.......................................................................................................................................................................................................................uuccaAagcuuucuugaac......................   1   1   JCK-1
.......................................................................................................................................................................................................................uuccaAagcuuucuugaac......................   3   1   LT-3
.......................................................................................................................................................................................................................uuccCcagcuuucuugaac......................   1   1   LCK-2
.......................................................................................................................................................................................................................uuccacagcuuucuugaaU......................   24   1   LCK-2
.......................................................................................................................................................................................................................uuccacCgcuuucuugaac......................   9   1   JCK-2
.......................................................................................................................................................................................................................uuccacCgcuuucuugaac......................   2   1   JCK-1
.......................................................................................................................................................................................................................uuccacagcuuucuugaac......................   6061   0   LT-1
.......................................................................................................................................................................................................................uuccacagcuuucGugaac......................   3   1   LCK-1
.......................................................................................................................................................................................................................uuccacagUuuucuugaac......................   3   1   LT-2
.......................................................................................................................................................................................................................uuccacagcuuucuugaUc......................   3   1   LT-2
.......................................................................................................................................................................................................................uNccacagcuuucuugaac......................   1   1   JCK-1
.......................................................................................................................................................................................................................uuUcacagcuuucuugaac......................   1   1   LT-3
.......................................................................................................................................................................................................................uuccacagcuuucuugaac......................   8638   0   LT-3
.......................................................................................................................................................................................................................uuccacagcuuucuugCac......................   3   1   JT-1
.......................................................................................................................................................................................................................uuccacagcuuucuugaGc......................   1   1   LCK-3
.......................................................................................................................................................................................................................uuccacagcuuucuugaCc......................   1   1   LCK-1
.......................................................................................................................................................................................................................uuccacagcuuucuugaac......................   2667   0   JCK-3
.......................................................................................................................................................................................................................uucAacagcuuucuugaac......................   1   1   LT-2
.......................................................................................................................................................................................................................uuccaUagcuuucuugaac......................   1   1   JCK-2
.......................................................................................................................................................................................................................uuccacagcuuucuugCac......................   3   1   LCK-3
.......................................................................................................................................................................................................................uuccCcagcuuucuugaac......................   2   1   LCK-1
.......................................................................................................................................................................................................................uuccacagcuuucuNgaac......................   4   1   LCK-1
.......................................................................................................................................................................................................................uuccacagcuuucuugCac......................   1   1   LCK-1
.......................................................................................................................................................................................................................uuccacagcuuucNugaac......................   6   1   LCK-1
.......................................................................................................................................................................................................................uCccacagcuuucuugaac......................   2   1   LT-3
.......................................................................................................................................................................................................................uuccacagcuuNcuugaac......................   2   1   JCK-1
.......................................................................................................................................................................................................................uuccacagcuCucuugaac......................   2   1   JCK-2
.......................................................................................................................................................................................................................uuccacagcuuucuugCac......................   1   1   LCK-2
.......................................................................................................................................................................................................................uuNcacagcuuucuugaac......................   1   1   LCK-1
.......................................................................................................................................................................................................................uuccacCgcuuucuugaac......................   2   1   JCK-3
.......................................................................................................................................................................................................................uuccacagcuuCcuugaac......................   3   1   LCK-3
.......................................................................................................................................................................................................................uuccacagcuuucuuAaac......................   4   1   LT-2
.......................................................................................................................................................................................................................uNccacagcuuucuugaac......................   3   1   JT-1
.......................................................................................................................................................................................................................uuccacagcuuucuugCac......................   11   1   LT-3
.......................................................................................................................................................................................................................uuccacagcuuucuugaaU......................   26   1   LCK-1
.......................................................................................................................................................................................................................uNccacagcuuucuugaac......................   5   1   JCK-2
.......................................................................................................................................................................................................................uuccacagcuuucuugCac......................   5   1   JCK-2
.......................................................................................................................................................................................................................uucUacagcuuucuugaac......................   4   1   JCK-2
.......................................................................................................................................................................................................................uuccCcagcuuucuugaac......................   16   1   LT-3
.......................................................................................................................................................................................................................uuccacagcuuucCugaac......................   2   1   LT-1
.......................................................................................................................................................................................................................uuccCcagcuuucuugaac......................   1   1   JT-3
.......................................................................................................................................................................................................................uucUacagcuuucuugaac......................   2   1   JCK-1
.......................................................................................................................................................................................................................Cuccacagcuuucuugaac......................   1   1   LT-2
.......................................................................................................................................................................................................................uuccacagUuuucuugaac......................   5   1   LT-1
.......................................................................................................................................................................................................................uuUcacagcuuucuugaac......................   1   1   LT-1
.......................................................................................................................................................................................................................uuccacagcuuuUuugaac......................   4   1   JCK-1
.......................................................................................................................................................................................................................uuccacagcuuuAuugaac......................   2   1   LCK-3
.......................................................................................................................................................................................................................uuNcacagcuuucuugaac......................   14   1   LT-2
.......................................................................................................................................................................................................................uuccacagcuuucuugaCc......................   5   1   LT-1
.......................................................................................................................................................................................................................uuccaAagcuuucuugaac......................   1   1   LCK-3
.......................................................................................................................................................................................................................uuccacCgcuuucuugaac......................   2   1   JT-1
.......................................................................................................................................................................................................................uuccacagcuuucuugaCc......................   2   1   LCK-2
.......................................................................................................................................................................................................................Cuccacagcuuucuugaac......................   3   1   LT-3
.......................................................................................................................................................................................................................uuccacagcuuucuGgaac......................   2   1   LCK-1
.......................................................................................................................................................................................................................uuccacCgcuuucuugaac......................   16   1   LT-3
.......................................................................................................................................................................................................................uuccacagcuuucuugaacC.....................   3   1   JT-3
.......................................................................................................................................................................................................................uuccacagcuuucuugaacA.....................   196   1   LCK-3
.......................................................................................................................................................................................................................uuccacagcuuucuugaacC.....................   18   1   LT-3
.......................................................................................................................................................................................................................uuccacagcuuCcuugaacu.....................   2   1   LT-3
.......................................................................................................................................................................................................................uuccacagcuuucuugaacu.....................   10755   0   LCK-3
.......................................................................................................................................................................................................................uuccacagcuCucuugaacu.....................   1   1   JT-1
.......................................................................................................................................................................................................................uuccacagcuuucGugaacu.....................   1   1   LCK-1
.......................................................................................................................................................................................................................uNccacagcuuucuugaacu.....................   2   1   JCK-3
.......................................................................................................................................................................................................................uuccacagcuuucuugaaUu.....................   192   1   LCK-3
.......................................................................................................................................................................................................................uuccacagcuuucuugaacA.....................   11   1   JT-3
.......................................................................................................................................................................................................................uuccacCgcuuucuugaacu.....................   4   1   LT-1
.......................................................................................................................................................................................................................uuccacagcuuNcuugaacu.....................   1   1   JT-2
```

```
                                                                                                                                                                 Star                                                              Mature
ucucuuucucucucucucucucuaaaaaaacaaaaggaaagaaaaaaaaaaaaaaaaaacuuacuacuacuacuaguaguauaucuucuaauuugcuggaaaggcacaccaaagagaaagaaauuaaaccaaggaaggaaggaaggaaggaaggaagugagaucugaugagagaaaaagagaauguuuuugcaaguccuggucaugcuuuuccacagcuuucuugaacuuucuuucuuucuguuuagucu
...............................................................................................................................................................................................................uuccacCgcuuucuugaacu.....................    1       1    JT-1
...............................................................................................................................................................................................................uuccacagcuuucuugaaUu.....................    132     1    LT-1
...............................................................................................................................................................................................................uuccacagcuuucuugaGcu.....................    3       1    JCK-1
...............................................................................................................................................................................................................uCccacagcuuucuugaacu.....................    1       1    LT-3
...............................................................................................................................................................................................................uNccacagcuuucuugaacu.....................    4       1    JCK-1
...............................................................................................................................................................................................................uuNcacagcuuucuugaacu.....................    33      1    LT-2
...............................................................................................................................................................................................................uuccacagcCuucuugaacu.....................    3       1    JCK-2
...............................................................................................................................................................................................................uuccacagcuuucuugaaAu.....................    1       1    JCK-1
...............................................................................................................................................................................................................uuccacagcuuucuugaaUu.....................    18      1    JT-1
...............................................................................................................................................................................................................uCccacagcuuucuugaacu.....................    3       1    JCK-3
...............................................................................................................................................................................................................uuccacagcuuucuugaacC.....................    2       1    LCK-1
...............................................................................................................................................................................................................uuccacagcuuucuugaacA.....................    20      1    JT-1
...............................................................................................................................................................................................................uuccacagcuuuNuugaacu.....................    1       1    LCK-1
...............................................................................................................................................................................................................uuccacagcuuucuugaaNu.....................    1       1    LT-1
...............................................................................................................................................................................................................uuccaAagcuuucuugaacu.....................    1       1    LCK-1
...............................................................................................................................................................................................................uCccacagcuuucuugaacu.....................    1       1    LT-1
...............................................................................................................................................................................................................uuccacagcuCucuugaacu.....................    2       1    LT-2
...............................................................................................................................................................................................................uuccacGgcuuucuugaacu.....................    1       1    LCK-3
...............................................................................................................................................................................................................uuccacagcuuuAuugaacu.....................    2       1    LT-3
...............................................................................................................................................................................................................uuccacagcuuucuugaGcu.....................    2       1    LCK-3
...............................................................................................................................................................................................................uuccCcagcuuucuugaacu.....................    2       1    JT-3
...............................................................................................................................................................................................................uuccacagcuuucuugaNcu.....................    1       1    LCK-1
...............................................................................................................................................................................................................uucUacagcuuucuugaacu.....................    3       1    LT-2
...............................................................................................................................................................................................................uuccacagcuuucuCgaacu.....................    1       1    LT-3
...............................................................................................................................................................................................................uuccacagcuuGcuugaacu.....................    1       1    JCK-1
...............................................................................................................................................................................................................uuccacagcuuucuugCacu.....................    2       1    LCK-2
...............................................................................................................................................................................................................Cuccacagcuuucuugaacu.....................    2       1    JCK-3
...............................................................................................................................................................................................................uuccaAagcuuucuugaacu.....................    3       1    LT-1
...............................................................................................................................................................................................................uuUcacagcuuucuugaacu.....................    1       1    JCK-3
...............................................................................................................................................................................................................uuccacagcuuucuugaacG.....................    2       1    LT-1
...............................................................................................................................................................................................................uuccacagcuuucuugGacu.....................    1       1    LT-3
...............................................................................................................................................................................................................uuccacagcCuucuugaacu.....................    1       1    JT-2
...............................................................................................................................................................................................................Cuccacagcuuucuugaacu.....................    1       1    LT-2
...............................................................................................................................................................................................................uuccacaAcuuucuugaacu.....................    1       1    LCK-2
...............................................................................................................................................................................................................uuccacagcCuucuugaacu.....................    1       1    LT-3
...............................................................................................................................................................................................................uuccacGgcuuucuugaacu.....................    2       1    JT-3
...............................................................................................................................................................................................................uuccaAagcuuucuugaacu.....................    1       1    JCK-2
...............................................................................................................................................................................................................uuccacagcuCucuugaacu.....................    3       1    LT-1
...............................................................................................................................................................................................................uuccacagcuuucuugaacu.....................    7087    0    LT-3
...............................................................................................................................................................................................................uuccacagcuuucuugaacC.....................    8       1    JT-1
...............................................................................................................................................................................................................uuccacagcuuucuugaaAu.....................    2       1    LT-3
...............................................................................................................................................................................................................uuccacaAcuuucuugaacu.....................    1       1    JT-1
...............................................................................................................................................................................................................uuccacagUuuucuugaacu.....................    1       1    LCK-3
...............................................................................................................................................................................................................uucUacagcuuucuugaacu.....................    3       1    LCK-3
...............................................................................................................................................................................................................uuccacagcuuucuugaaUu.....................    172     1    LT-3
...............................................................................................................................................................................................................uuccacCgcuuucuugaacu.....................    2       1    LCK-1
...............................................................................................................................................................................................................uuccacagcuuucuugaCcu.....................    8       1    LT-3
...............................................................................................................................................................................................................uuccacagcuuucuugaacA.....................    48      1    JCK-1
...............................................................................................................................................................................................................uuccUcagcuuucuugaacu.....................    1       1    JCK-2
...............................................................................................................................................................................................................uuccacagcuuucuugaCcu.....................    3       1    JCK-2
...............................................................................................................................................................................................................uuccacagcuuucuugaacC.....................    39      1    LT-2
...............................................................................................................................................................................................................uuccacagcuuucuugaaUu.....................    111     1    JCK-2
...............................................................................................................................................................................................................uuccacaAcuuucuugaacu.....................    1       1    LT-2
...............................................................................................................................................................................................................uuccacagcuuucuugaacu.....................    2326    0    LCK-2
...............................................................................................................................................................................................................uuccacagcuuucuugGacu.....................    1       1    LT-2
...............................................................................................................................................................................................................uuccacagcuuucuugaCcu.....................    1       1    JCK-3
...............................................................................................................................................................................................................uuccacagcuuucuGgaacu.....................    1       1    LT-3
...............................................................................................................................................................................................................uuccacagcuuucuugaCcu.....................    1       1    JT-2
...............................................................................................................................................................................................................uuccacagcuuucuugaacA.....................    172     1    LT-3
...............................................................................................................................................................................................................uuccacagcuuucuugaacC.....................    41      1    LCK-3
...............................................................................................................................................................................................................uuccCcagcuuucuugaacu.....................    3       1    LT-1
...............................................................................................................................................................................................................uuccacaAcuuucuugaacu.....................    4       1    LCK-3
...............................................................................................................................................................................................................uCccacagcuuucuugaacu.....................    2       1    JCK-1
...............................................................................................................................................................................................................uuccacagUuuucuugaacu.....................    1       1    JT-1
...............................................................................................................................................................................................................uuccacagcuuCcuugaacu.....................    1       1    JCK-2
...............................................................................................................................................................................................................uuccGcagcuuucuugaacu.....................    1       1    LCK-3
...............................................................................................................................................................................................................uuccacagcuuucuugaaUu.....................    50      1    JT-3
...............................................................................................................................................................................................................uuccacagcuuucuugaacu.....................    4415    0    LT-1
...............................................................................................................................................................................................................uuccacagcuuucuugaCcu.....................    3       1    JCK-1
...............................................................................................................................................................................................................uuccacagcuuucCugaacu.....................    1       1    JT-1
```

Star Mature

ucucuuucucucucucucucucucuaaaaaaaacaaaaggaaagaaaaaaaaaaaaaaaaacuuacuacuacuacuaguaguauaucuucuaauuugcuggaaaggcacaccaaagagaaagaaauuaaaccaaggaaggaaggaaggaaggaaggaagugagaucugaugagagaaaaagagaauguuuuugcaaguccuggucaugcuuuuccacagcuuucuugaacuuucuuucuuucuguuuagucu

..........uuUcacagcuuucuugaacu..........	4	1	JCK-2
..........uuccacCgcuuucuugaacu..........	8	1	LT-3
..........uuccacagcuuucuugaaUu..........	24	1	LCK-1
..........uuccacagcuuucCugaacu..........	1	1	LT-3
..........uuccacagcuuucuugaaUu..........	52	1	JCK-3
..........uuccacagcCuucuugaacu..........	5	1	LT-1
..........uuccacagcuuucuugaacC..........	3	1	JT-2
..........uNccacagcuuucuugaacu..........	1	1	JT-1
..........uucNacagcuuucuugaacu..........	2	1	JCK-1
..........uuccacagcuuucuugaacA..........	43	1	JCK-3
..........uuccacagcuuucuugaacu..........	6887	0	LT-2
..........uuccacagcuuucuugaacA..........	87	1	LT-1
..........uuccaAagcuuucuugaacu..........	3	1	LT-3
..........uuccacagcuuucuugaacu..........	2055	0	JT-1
..........uuccacagcuuucuuAaacu..........	3	1	LCK-3
..........uuccacagcCuucuugaacu..........	7	1	LT-2
..........uuccacagcuuucCugaacu..........	1	1	JCK-2
..........uuccacagcuuucNugaacu..........	5	1	LCK-1
..........uuccacagcuuucuugaaUu..........	45	1	JT-2
..........uuccCcagcuuucuugaacu..........	1	1	JCK-2
..........uuccacagcuuucuugaacu..........	731	0	JT-3
..........uuccacagcuuucuugaCcu..........	1	1	LT-1
..........uCccacagcuuucuugaacu..........	1	1	LCK-3
..........uuccacagcuuucuugaacG..........	2	1	JCK-2
..........uuccacagcuuucCugaacu..........	1	1	LT-1
..........uuccacagcuuucuugaacu..........	2316	0	LCK-1
..........uuccUcagcuuucuugaacu..........	1	1	JCK-1
..........uuccacagcuuucuGgaacu..........	4	1	LCK-1
..........uuccacagcuuucuugaacG..........	45	1	LCK-3
..........uuccacCgcuuucuugaacu..........	3	1	LCK-2
..........uuccacagcuuucuugaCcu..........	11	1	LCK-3
..........uuccacagcuuucuugaNcu..........	2	1	JCK-3
..........uuccaNagcuuucuugaacu..........	3	1	LCK-1
..........Cuccacagcuuucuugaacu..........	2	1	LCK-1
..........uuccacagcuuuAuugaacu..........	4	1	LCK-3
..........uuccCcagcuuucuugaacu..........	1	1	LCK-1
..........uuccCcagcuuucuugaacu..........	8	1	LT-3
..........uuccacagcuuuUuugaacu..........	2	1	LT-1
..........uCccacagcuuucuugaacu..........	2	1	JCK-2
..........uuccaUagcuuucuugaacu..........	3	1	JCK-2
..........uuccacCgcuuucuugaacu..........	3	1	JCK-3
..........uuccacagcuuucuugaaUu..........	154	1	LT-2
..........uuccacagcuuucuuAaacu..........	1	1	JCK-1
..........uuccacagcuuucuNgaacu..........	7	1	LCK-1
..........uuccacagcuuucuugaacA..........	2	1	LCK-1
..........uuccacagcuuucuugaacA..........	57	1	JCK-2
..........uuccacagcuuucuGgaacu..........	1	1	JT-1
..........uuccacagUuuucuugaacu..........	1	1	LT-1
..........uuccacagcuGucuugaacu..........	1	1	LT-2
..........Cuccacagcuuucuugaacu..........	2	1	LT-1
..........uuccacagcuuucuugaaUu..........	35	1	LCK-2
..........uNccacagcuuucuugaacu..........	1	1	JCK-2
..........uuccacagcuuucuugaacu..........	2309	0	JCK-3
..........uuccacagcuuucGugaacu..........	1	1	LCK-2
..........uuccacagcuuCcuugaacu..........	2	1	LT-2
..........uuccacagcuuucuugaaAu..........	6	1	LCK-3
..........uuccacagcuuucuugaacu..........	2125	0	JCK-1
..........uuccacagUuuucuugaacu..........	1	1	JCK-2
..........Cuccacagcuuucuugaacu..........	4	1	LCK-3
..........uuccacagcuuucuugCacu..........	2	1	LCK-1
..........uuccacagcuuucuuUaacu..........	1	1	LT-2
..........uuccacagcuuucuCgaacu..........	1	1	JCK-1
..........uuccacagcuuucuugaacC..........	2	1	JCK-2
..........uuccacaUcuuucuugaacu..........	1	1	JCK-1
..........uuccacagcuuNcuugaacu..........	3	1	JCK-1
..........uuccacagcuuucuugaaAu..........	2	1	LT-1
..........uucUacagcuuucuugaacu..........	1	1	JCK-1
..........uuccacagcuuNcuugaacu..........	3	1	JT-1
..........uuccacCgcuuucuugaacu..........	6	1	JCK-2
..........uuccaAagcuuucuugaacu..........	1	1	JCK-3

```
                                                                                                                                                                                                    Star                                                               Mature
ucucuuucucucucucucucucucuaaaaaaaacaaaaggaaagaaaaaaaaaaaaaaaaaacuuacuacuacuacuaguaguauaucuucuaauuugcuggaaaggcacaccaaagagaaagaaauuaaaccaaggaaggaaggaaggaaggaaggaagugagaucugaugagagaaaaagagaauguuuuugcaaguccuggucaugcuuuuccacagcuuucuugaacuuucuuucuuucuguuuagucu
....................................................................................................................................................................................................................uuccCcagcuuucuugaacu.....................   2      1   JT-1
....................................................................................................................................................................................................................Cuccacagcuuucuugaacu.....................   1      1   JCK-2
....................................................................................................................................................................................................................Cuccacagcuuucuugaacu.....................   1      1   LT-3
....................................................................................................................................................................................................................uuccacagcuuucuugCacu.....................   2      1   JCK-3
....................................................................................................................................................................................................................uuccacagcuuucuugaCcu.....................   1      1   LCK-2
....................................................................................................................................................................................................................uuccCcagcuuucuugaacu.....................   8      1   LCK-3
....................................................................................................................................................................................................................uuccacagcuuucuugaUcu.....................   2      1   LT-2
....................................................................................................................................................................................................................uuccacagcuGucuugaacu.....................   1      1   JCK-1
....................................................................................................................................................................................................................uuccacagcuuucuugCacu.....................   3      1   JCK-2
....................................................................................................................................................................................................................uuccacagcuuucuugaaAu.....................   1      1   JCK-3
....................................................................................................................................................................................................................uuccacagcuuucuugaCcu.....................   3      1   JT-1
....................................................................................................................................................................................................................uuccacagcuuuAuugaacu.....................   2      1   JCK-3
....................................................................................................................................................................................................................uuccacagcuuucuugCacu.....................   10     1   LT-2
....................................................................................................................................................................................................................uuccacagcuuucuuCaacu.....................   1      1   JCK-1
....................................................................................................................................................................................................................uuccacagcuuucuugaacG.....................   8      1   LT-2
....................................................................................................................................................................................................................uuccacagcuuucuugCacu.....................   6      1   LT-3
....................................................................................................................................................................................................................uuccacagcuuucuugaaAu.....................   6      1   LT-2
....................................................................................................................................................................................................................uuccacaAcuuucuugaacu.....................   1      1   LT-3
....................................................................................................................................................................................................................uuccacagcuuucuugaaUu.....................   72     1   JCK-1
....................................................................................................................................................................................................................uuccacagcuuucuuAaacu.....................   1      1   LT-2
....................................................................................................................................................................................................................uuccaAagcuuucuugaacu.....................   2      1   JCK-1
....................................................................................................................................................................................................................uuccacagcuuucuugaacG.....................   6      1   LT-3
....................................................................................................................................................................................................................uuccCcagcuuucuugaacu.....................   20     1   LT-2
....................................................................................................................................................................................................................uuccacagcuuucuugGacu.....................   8      1   LCK-3
....................................................................................................................................................................................................................uuccacagcuuucuugaacA.....................   137    1   LT-2
....................................................................................................................................................................................................................uuccacagcuuucuugaacC.....................   9      1   LT-1
....................................................................................................................................................................................................................uuccacagcuuuUuugaacu.....................   1      1   LT-3
....................................................................................................................................................................................................................uuccacagcuuucuugaacG.....................   1      1   JT-1
....................................................................................................................................................................................................................uuccacagcuuucuugaCcu.....................   25     1   LT-2
....................................................................................................................................................................................................................uuccaAagcuuucuugaacu.....................   7      1   LCK-3
....................................................................................................................................................................................................................uuccacagcuuucuugaacu.....................   544    0   JT-2
....................................................................................................................................................................................................................uuccacagcuuCcuugaacu.....................   1      1   LCK-3
....................................................................................................................................................................................................................uuccacaAcuuucuugaacu.....................   1      1   LT-1
....................................................................................................................................................................................................................uuccacagcuuucuugaacu.....................   3389   0   JCK-2
....................................................................................................................................................................................................................uuccacagcuuucuugCacu.....................   10     1   LT-1
....................................................................................................................................................................................................................uuccacagcuuucuugaacA.....................   3      1   JT-2
....................................................................................................................................................................................................................uuccacagcuCucuugaacu.....................   1      1   LCK-3
....................................................................................................................................................................................................................uuccacagcuuucuugaCcu.....................   3      1   LCK-1
....................................................................................................................................................................................................................uuccacagcuuucuugaacC.....................   5      1   JCK-3
....................................................................................................................................................................................................................uuccacagcCuucuugaacu.....................   1      1   JT-3
....................................................................................................................................................................................................................uuGcacagcuuucuugaacu.....................   5      1   LT-2
....................................................................................................................................................................................................................uuNcacagcuuucuugaacu.....................   2      1   LCK-3
....................................................................................................................................................................................................................uuccacCgcuuucuugaacu.....................   9      1   LCK-3
....................................................................................................................................................................................................................uuccacagcuuucuCgaacu.....................   1      1   LT-2
....................................................................................................................................................................................................................uuccacagcuuucCugaacu.....................   2      1   LCK-3
....................................................................................................................................................................................................................uuccacCgcuuucuugaacu.....................   3      1   JCK-1
....................................................................................................................................................................................................................uuccacagcuuuAuugaacu.....................   1      1   LT-2
....................................................................................................................................................................................................................uuccacagcuuucuugaUcu.....................   2      1   LT-1
....................................................................................................................................................................................................................uNccacagcuuucuugaacu.....................   2      1   LCK-1
....................................................................................................................................................................................................................uuccacagcCuucuugaacu.....................   1      1   JCK-1
....................................................................................................................................................................................................................uuccacagcuuucuugaacC.....................   7      1   JCK-1
....................................................................................................................................................................................................................uuccCcagcuuucuugaacu.....................   4      1   JCK-1
....................................................................................................................................................................................................................uuccacCgcuuucuugaacu.....................   13     1   LT-2
....................................................................................................................................................................................................................uuccacagcuuucuugCacu.....................   10     1   LCK-3
....................................................................................................................................................................................................................uuccacagcuuucuugCacu.....................   3      1   JCK-1
....................................................................................................................................................................................................................uuccacagcuuucuugaacN.....................   2      1   JCK-1
....................................................................................................................................................................................................................uuccacagcCuucuugaacu.....................   1      1   JCK-3
....................................................................................................................................................................................................................uuccacagcuuucuugaacuC....................   53     1   JCK-1
....................................................................................................................................................................................................................uuccacagcuuucuugaacuA....................   5      1   JT-3
....................................................................................................................................................................................................................uuccacCgcuuucuugaacuu....................   1      1   JT-1
....................................................................................................................................................................................................................uuccacagcuuucuugaacuu....................   191    0   LCK-2
....................................................................................................................................................................................................................uuccaAagcuuucuugaacuu....................   1      1   LT-2
....................................................................................................................................................................................................................uuccacagcuuucuugaaUuu....................   88     1   JCK-2
....................................................................................................................................................................................................................uuccacagcuuucuugaacCu....................   1      1   LT-2
....................................................................................................................................................................................................................uCccacagcuuucuugaacuu....................   1      1   LCK-3
....................................................................................................................................................................................................................uuccacagcuuucuugaacuA....................   67     1   JCK-3
....................................................................................................................................................................................................................uuccacagcuuucuugaacuC....................   6      1   LCK-1
....................................................................................................................................................................................................................Cuccacagcuuucuugaacuu....................   1      1   LT-3
....................................................................................................................................................................................................................uCccacagcuuucuugaacuu....................   1      1   JCK-2
....................................................................................................................................................................................................................uuccacagcuuucuugaaUuu....................   112    1   LCK-3
```

Star / Mature<br>ucucuuucucucucucucucucucuaaaaaaaacaaaaggaaagaaaaaaaaaaaaaaaaaaacuuacuacuacuacuaguaguauaucuucuaauuugcuggaaaggcacaccaaagagaaagaaauuaaaccaaggaaggaaggaaggaaggaaggaagugagaucugaugagagaaaaagagaauguuuuugcaaguccuggucaugcuuuuccacagcuuucuugaacuuucuuucuuucuguuuagucu			
...uuccacagcuuucuugCacuu...	1	1	LT-1
...uuccacagcuuuUuugaacuu...	1	1	LT-1
...uuccacagcuuucuugaacGu...	1	1	JCK-2
...uuccacGgcuuucuugaacuu...	7	1	JT-2
...uuccacagcuuucuugaacuA...	20	1	LCK-1
...uuccacagcuuucuugaacuC...	68	1	JCK-3
...uNccacagcuuucuugaacuu...	1	1	JT-1
...uuccacagcuuucuugaacuA...	23	1	LCK-2
...uuccacagcuuucuugaCcuu...	2	1	JT-1
...uuccacagcuuucuugCacuu...	2	1	LT-2
...uuccacagcuuucuugaaUuu...	61	1	JCK-3
...uuccacagcuuucuugCacuu...	2	1	JCK-3
...uuccacagcuuucuugaacAu...	8	1	LCK-3
...uuccacagcuuucuugaaAuu...	5	1	LT-2
...uuccacagcuuuUuugaacuu...	1	1	JCK-2
...uuccacagcuuucuugaacuu...	2349	0	JCK-1
...uNccacagcuuucuugaacuu...	1	1	LT-2
...uuccacagcuuucuugaacuC...	53	1	LCK-3
...uuUcacagcuuucuugaacuu...	2	1	JCK-3
...uuccacagcuuucuugaacAu...	6	1	LT-2
...uNccacagcuuucuugaacuu...	1	1	JCK-1
...uuNcacagcuuucuugaacuu...	4	1	LT-2
...uuccacagcuuucuugaacuA...	66	1	LT-1
...uuccacagcuuucuugaCcuu...	3	1	LCK-3
...uuccacagcuCucuugaacuu...	1	1	JT-2
...Cuccacagcuuucuugaacuu...	3	1	JCK-3
...uuccacagcuuucuugCacuu...	2	1	LT-3
...uuccacagcuuuAuugaacuu...	1	1	LT-3
...uuccCcagcuuucuugaacuu...	2	1	JCK-3
...uuccaUagcuuucuugaacuu...	3	1	JCK-3
...uuccacagcuuucuugaacNu...	2	1	JCK-1
...uuccacCgcuuucuugaacuu...	3	1	LCK-3
...uuccacagcuuucuugaCcuu...	1	1	LT-3
...uuccacagcuuucuugaacuC...	44	1	LT-2
...uuccacagcuuucuugaacuC...	18	1	JT-1
...uuccacagcuuucCugaacuu...	1	1	JCK-1
...uuccacagcuuucuugaaAuu...	1	1	LT-1
...uuccaUagcuuucuugaacuu...	1	1	JCK-1
...uuccCcagcuuucuugaacuu...	5	1	LT-2
...uuccacagcuuucuugaCcuu...	3	1	JCK-1
...uuccacagcuuucuugaaUuu...	29	1	LCK-1
...uuccacagcuuucuugaacuu...	145	0	JT-3
...uuccacagcuuuAuugaacuu...	3	1	JCK-3
...uuccacagcuuucuugaacAu...	1	1	LT-1
...uuccacagcuuucuugCacuu...	1	1	JT-1
...uuccacagcuuucuugaacGu...	1	1	LT-1
...uuccacaAcuuucuugaacuu...	1	1	LT-2
...uuccacagcuuucuugaacuA...	240	1	LCK-3
...uuUcacagcuuucuugaacuu...	2	1	JT-2
...uuccacGgcuuucuugaacuu...	1	1	JCK-2
...uuccacagcuuucuugaaUuu...	11	1	JT-3
...uuUcacagcuuucuugaacuu...	1	1	JT-3
...uuccacagAuuucuugaacuu...	1	1	JCK-1
...uNccacagcuuucuugaacuu...	4	1	JCK-2
...uucGacagcuuucuugaacuu...	1	1	JCK-1
...uuccacagcuuuAuugaacuu...	1	1	LCK-3
...uucNacagcuuucuugaacuu...	1	1	JCK-3
...uucUacagcuuucuugaacuu...	4	1	JCK-2
...uuccacagcuuucuuAaacuu...	1	1	LT-1
...uuccacagcuuucuugaacuu...	1769	0	LT-3
...uuccaAagcuuucuugaacuu...	3	1	JCK-3
...uuccacagcuuucuugaCcuu...	4	1	LT-2
...uuccaNagcuuucuugaacuu...	2	1	LCK-1
...uuccacagcuuucuugaaUuu...	101	1	LT-2
...uuccacCgcuuucuugaacuu...	3	1	JCK-3
...uuccacCgcuuucuugaacuu...	5	1	JCK-1
...uuccacagcuuucuugCacuu...	5	1	JCK-2
...uuccacagcuuucCugaacuu...	1	1	JCK-2
...uuccacagcuuucuugaacuu...	2427	0	LCK-3
...uuccacagcuuCcuugaacuu...	1	1	LT-2

```
                                                                                                                                                                                  Star                                                                Mature
ucucuuucucucucucucucucuaaaaaaacaaaaggaaagaaaaaaaaaaaaaaaaaaacuuacuacuacuacuaguaguauaucuucuaauuugcuggaaaggcacaccaaagagaaagaaauuaaaccaaggaaggaaggaaggaaggaaggaagugagaucugaugagagaaaaagagaauguuuuugcaaguccuggucaugcuuuuccacagcuuucuugaacuuucuuucuuucuguuuagucu
..................................................................................................................................................................................................................uuccacagcuuucuugaGcuu....................    4     1   JCK-1
..................................................................................................................................................................................................................uuccaAagcuuucuugaacuu....................    2     1   JCK-1
..................................................................................................................................................................................................................uuccacagcuuucuugaacuu....................    1431  0   LT-2
..................................................................................................................................................................................................................uuccacagcuuucuuAaacuu....................    1     1   LCK-2
..................................................................................................................................................................................................................uuccacCgcuuucuugaacuu....................    3     1   JCK-2
..................................................................................................................................................................................................................uuccacagcuuucuuAaacuu....................    1     1   JCK-1
..................................................................................................................................................................................................................uCccacagcuuucuugaacuu....................    1     1   LT-2
..................................................................................................................................................................................................................uuccacagcuuucuugCacuu....................    2     1   JCK-1
..................................................................................................................................................................................................................uuccacagcuuucuugaaUuu....................    86    1   LT-1
..................................................................................................................................................................................................................uuccacagcuuucuugaacAu....................    3     1   JCK-3
..................................................................................................................................................................................................................uuccacagcuuuAuugaacuu....................    1     1   LT-2
..................................................................................................................................................................................................................uuccCcagcuuucuugaacuu....................    6     1   JCK-2
..................................................................................................................................................................................................................uuUcacagcuuucuugaacuu....................    6     1   JCK-2
..................................................................................................................................................................................................................uuccacaAcuuucuugaacuu....................    1     1   LCK-3
..................................................................................................................................................................................................................uNccacagcuuucuugaacuu....................    6     1   JCK-3
..................................................................................................................................................................................................................uuccacagcuuucuugaacuA....................    148   1   LT-3
..................................................................................................................................................................................................................uuccacagcuuucuugaacuA....................    2     1   JT-2
..................................................................................................................................................................................................................uuccacagcuuucuugaacuA....................    134   1   LT-2
..................................................................................................................................................................................................................uuccCcagcuuucuugaacuu....................    1     1   LCK-3
..................................................................................................................................................................................................................uuccacagcuuucuCgaacuu....................    5     1   LT-2
..................................................................................................................................................................................................................uuccacagcuuucuugaCcuu....................    5     1   JCK-2
..................................................................................................................................................................................................................uucUacagcuuucuugaacuu....................    1     1   JCK-3
..................................................................................................................................................................................................................uuccaUagcuuucuugaacuu....................    1     1   LT-3
..................................................................................................................................................................................................................uuccacagcuuucuugaacuC....................    9     1   LCK-2
..................................................................................................................................................................................................................uuccacGgcuuucuugaacuu....................    11    1   JT-3
..................................................................................................................................................................................................................uuccacagcCuucuugaacuu....................    2     1   LT-1
..................................................................................................................................................................................................................uuccacagcuuucuugaaUuu....................    135   1   LT-3
..................................................................................................................................................................................................................uuccacagcuuucuugaacAu....................    2     1   JCK-1
..................................................................................................................................................................................................................uuccacagNuuucuugaacuu....................    1     1   JCK-3
..................................................................................................................................................................................................................uuccacagcuuucuugaacAu....................    5     1   JCK-2
..................................................................................................................................................................................................................uuccCcagcuuucuugaacuu....................    2     1   JCK-1
..................................................................................................................................................................................................................uuccacagcuuucuugaacGu....................    1     1   LT-3
..................................................................................................................................................................................................................uuccaAagcuuucuugaacuu....................    5     1   JCK-2
..................................................................................................................................................................................................................uuccacagcuuucuugCacuu....................    1     1   LCK-3
..................................................................................................................................................................................................................uuccacagcuuucuugaacuu....................    157   0   LCK-1
..................................................................................................................................................................................................................uuccacagcuuucuugaacuC....................    33    1   LT-1
..................................................................................................................................................................................................................uuccCcagcuuucuugaacuu....................    3     1   LT-3
..................................................................................................................................................................................................................uuccacagcuuucuugaacuu....................    502   0   JT-1
..................................................................................................................................................................................................................uuccacagcuuucuugaacuu....................    3888  0   JCK-2
..................................................................................................................................................................................................................uuccacagcuuucuugaacuu....................    97    0   JT-2
..................................................................................................................................................................................................................uucNacagcuuucuugaacuu....................    2     1   JCK-1
..................................................................................................................................................................................................................uuccacagcuuucuugaaUuu....................    50    1   JCK-1
..................................................................................................................................................................................................................uuccacaAcuuucuugaacuu....................    1     1   LT-1
..................................................................................................................................................................................................................uuccacagcuuNcuugaacuu....................    3     1   JCK-1
..................................................................................................................................................................................................................uuccacagcuuucuugaacuC....................    54    1   LT-3
..................................................................................................................................................................................................................uuccacagcuuucuugaacuC....................    64    1   JCK-2
..................................................................................................................................................................................................................uuccacagcuuucuugaacuu....................    954   0   LT-1
..................................................................................................................................................................................................................uuccacagcuuucuugaacuA....................    75    1   JCK-1
..................................................................................................................................................................................................................uuccacagcuuucuugaaUuu....................    48    1   LCK-2
..................................................................................................................................................................................................................uuccacCgcuuucuugaacuu....................    3     1   LT-3
..................................................................................................................................................................................................................uuccacCgcuuucuugaacuu....................    1     1   LCK-2
..................................................................................................................................................................................................................uuccacagcuuucuugaacAu....................    6     1   LT-3
..................................................................................................................................................................................................................uuUcacagcuuucuugaacuu....................    1     1   JCK-1
..................................................................................................................................................................................................................uuccacagcuuucuugaacuA....................    125   1   JCK-2
..................................................................................................................................................................................................................uCccacagcuuucuugaacuu....................    1     1   LCK-2
..................................................................................................................................................................................................................uuccacagcuuucuugaacuA....................    97    1   JT-1
..................................................................................................................................................................................................................uuccacaAcuuucuugaacuu....................    1     1   JCK-3
..................................................................................................................................................................................................................uuccacagcuuucuugaacuu....................    2955  0   JCK-3
..................................................................................................................................................................................................................uuccacagcuuucuugaacuN....................    4     1   JCK-1
..................................................................................................................................................................................................................uuccacagcuuucuugaCcuu....................    2     1   JCK-3
..................................................................................................................................................................................................................uuccacagcuuucuugaaUuu....................    3     1   JT-2
..................................................................................................................................................................................................................uuccacagcuuucuugaaAuu....................    3     1   LCK-3
..................................................................................................................................................................................................................uuccacagcuuucCugaacuu....................    1     1   LT-3
..................................................................................................................................................................................................................uucNacagcuuucuugaacuu....................    1     1   JT-1
..................................................................................................................................................................................................................uuccacGgcuuucuugaacuu....................    1     1   JCK-1
..................................................................................................................................................................................................................uuccacagcCuucuugaacuu....................    1     1   JCK-3
..................................................................................................................................................................................................................uuccacagcuuucuuAaacuu....................    2     1   LCK-3
..................................................................................................................................................................................................................uuccacCgcuuucuugaacuuu...................    8     1   LT-2
..................................................................................................................................................................................................................uuccacagcuuucuugaaUuuu...................    27    1   JCK-1
..................................................................................................................................................................................................................uuccacagcuuucuugaacuuA...................    128   1   JCK-2
```

Star Mature

ucucuuucucucucucucucucucuaaaaaaaacaaaaggaaagaaaaaaaaaaaaaaaaaacuuacuacuacuacuaguaguauaucuucuaauuugcuggaaaggcacaccaaagagaaagaaauuaaaccaaggaaggaaggaaggaaggaaggaagugagaucugaugagagaaaaagagaauguuuuugcaaguccuggucaugcuuuuccacagcuuucuugaacuuucuuucuuucuguuuagucu

uCccacagcuuucuugaacuuu	3	1	LT-3
uuccacagcuuucuCgaacuuu	3	1	LT-3
uuccacaAcuuucuugaacuuu	2	1	LT-2
uuccacCgcuuucuugaacuuu	1	1	LCK-3
uuccacagcuuucuugaacCuu	3	1	JCK-2
uuUcacagcuuucuugaacuuu	7	1	LT-2
uuccacagcuuucuugaaUuuu	22	1	LCK-1
uuUcacagcuuucuugaacuuu	1	1	JCK-2
uuccGcagcuuucuugaacuuu	1	1	LT-2
uuccacagcuuucuugaacAuu	12	1	LT-1
uuccacagcuuucuugaacAuu	36	1	JCK-2
uuccacCgcuuucuugaacuuu	2	1	JCK-2
uCccacagcuuucuugaacuuu	1	1	LT-1
uuccacagcuuucuugaacuuu	1496	0	JCK-2
uuccacCgcuuucuugaacuuu	2	1	LT-1
uuccacagcuuucuugaacuuA	62	1	LT-1
uuccacagcuuucuugCacuuu	1	1	LCK-2
uuccacagcuuucuuUaacuuu	1	1	LT-3
uuccacagcuuucuugaacAuu	14	1	JCK-1
uuccacagcuuucuugaaAuuu	2	1	JCK-3
uuccacagcuuucuugaacuCu	6	1	JCK-2
uuccacagcuuucuugaacAuu	12	1	JCK-3
uuccacagcuuucuugaacCuu	1	1	LT-3
uuccCcagcuuucuugaacuuu	1	1	JCK-1
uuccacagcuuucuugaacuCu	5	1	JCK-3
uuccacagcuuucuugaacuAu	11	1	LT-1
uuccacagcuuucuugaacuuu	1432	0	LT-2
uuccacagcuuucuugaacAuu	16	1	LCK-3
uuccacagcuuucuugCacuuu	1	1	JCK-3
uNccacagcuuucuugaacuuu	1	1	JCK-1
uuccacCgcuuucuugaacuuu	1	1	JCK-1
uuccacagcuuNcuugaacuuu	3	1	JCK-1
uuccacagcuuucuugaacuuC	12	1	JT-3
uGccacagcuuucuugaacuuu	1	1	LCK-3
uuccacagcuuucuugaacuuu	64	0	JT-3
uuccacagcuuucuugaacuuN	1	1	LT-2
uuccacagcuuucuugaaUuuu	32	1	LT-3
uuccacagcuuucuugaacAuu	6	1	JT-2
uuccCcagcuuucuugaacuuu	3	1	LT-2
uuccacagcuuucuugaacuAu	12	1	LT-3
uuccacagcuuucuugaacAuu	29	1	LT-3
uuccacagcuuucuugaCcuuu	1	1	LCK-3
uuccCcagcuuucuugaacuuu	1	1	JCK-3
uNccacagcuuucuugaacuuu	1	1	JCK-3
uuccacagcuuucuugaaUuuu	41	1	LT-1
uuccacagcuuucuugaacuuA	49	1	JT-1
uuccacagcuuucuugaacuuA	157	1	LCK-3
uuccacagcuuucuugaacuuA	121	1	LT-2
uuccacagcuuuAuugaacuuu	1	1	LT-1
uuccacagcuuucuugaacuuC	114	1	LT-2
uuccacagcuuucuugaacuuu	1068	0	LT-1
uuccacagcuuucuugaacuuC	65	1	JCK-1
uuccacagcuuucuugaacGuu	1	1	LT-3
uuccacagcuuucuugaaUuuu	39	1	LCK-3
uuGcacagcuuucuugaacuuu	2	1	LT-2
uuccacagcuuucuugaacuuu	899	0	JCK-1
uuUcacagcuuucuugaacuuu	1	1	JCK-1
uuccacCgcuuucuugaacuuu	1	1	JT-1
uuccacagcuuucCugaacuuu	1	1	JCK-2
uuccCcagcuuucuugaacuuu	4	1	LCK-3
uuccacagcuuucuugaaAuuu	2	1	LT-3
uuccacagcuuucuugaacuuC	118	1	LT-3
uuccacagcuuucuugaacuCu	3	1	LT-3
uuccacagcuuucuugaacuuN	1	1	JCK-1
uuccacagcuuucuugaacuAu	6	1	JCK-3
uuccacagcuuucuugaacAuu	26	1	LT-2
uuccacCgcuuucuugaacuuu	2	1	LT-3
Cuccacagcuuucuugaacuuu	1	1	JCK-3
uuccacagcuuucuugaacuCu	5	1	LCK-3
uuccacagcuuucuugaaUuuu	7	1	JT-1

```
                                                                                                                                                    Star                                                          Mature
ucucuuucucucucucucucucuaaaaaaaacaaaaggaaagaaaaaaaaaaaaaaaaacuuacuacuacuacuaguaguauaucuucuaauuugcuggaaaggcacaccaaagagaaagaaauuaaaccaaggaaggaaggaaggaaggaaggaagugagaucugaugagagaaaaagagaauguuuuugcaaguccuggucaugcuuuuccacagcuuucuugaacuuucuuucuuucuguuuagucu
.................................................................................................................................................................................................................uuccacagcuuucuugaacuNu...................	1	1	JCK-1
.................................................................................................................................................................................................................uuccacagcCuucuugaacuuu...................	2	1	JCK-2
.................................................................................................................................................................................................................uuccacagcuuucuugaCcuuu...................	3	1	LT-2
.................................................................................................................................................................................................................uuccacagcuuucuugaCcuuu...................	2	1	LT-1
.................................................................................................................................................................................................................uuccacaAcuuucuugaacuuu...................	1	1	JT-3
.................................................................................................................................................................................................................uuccacagcuuucuugaacuuG...................	7	1	LT-2
.................................................................................................................................................................................................................uuccacCgcuuucuugaacuuu...................	3	1	JCK-3
.................................................................................................................................................................................................................uuccacagcuuucuugaaUuuu...................	37	1	LT-2
.................................................................................................................................................................................................................uuccacCgcuuucuugaacuuu...................	2	1	LCK-2
.................................................................................................................................................................................................................uuccacagcuuucuugaacuuA...................	17	1	JT-3
.................................................................................................................................................................................................................uuccacagcuGucuugaacuuu...................	2	1	JCK-1
.................................................................................................................................................................................................................uuccacagcuuucuugaaAuuu...................	2	1	JT-3
.................................................................................................................................................................................................................uuccacagcuuucuugaaUuuu...................	36	1	JCK-3
.................................................................................................................................................................................................................uuccacagcuuucuugaacuuA...................	21	1	LCK-1
.................................................................................................................................................................................................................uuccacagcuuucuugaacCuu...................	3	1	JCK-3
.................................................................................................................................................................................................................uuccacagcuuucuugCacuuu...................	1	1	LCK-3
.................................................................................................................................................................................................................uuccacagcuuucuugaaUuuu...................	3	1	JT-2
.................................................................................................................................................................................................................uucNacagcuuucuugaacuuu...................	1	1	JCK-1
.................................................................................................................................................................................................................uuccacagcuuucuugaacuuA...................	77	1	JCK-3
.................................................................................................................................................................................................................uuccacagcuuucuugaCcuuu...................	2	1	LT-3
.................................................................................................................................................................................................................uuccacagcuuucuugaacuAu...................	13	1	LT-2
.................................................................................................................................................................................................................uNccacagcuuucuugaacuuu...................	1	1	JCK-2
.................................................................................................................................................................................................................uuccacagcuuucuugaacuuC...................	55	1	JCK-3
.................................................................................................................................................................................................................uuccacagcuuucuugaacCuu...................	1	1	JCK-1
.................................................................................................................................................................................................................uuccacagcuuucuugaacuuG...................	2	1	JCK-2
.................................................................................................................................................................................................................uuccacagcuuucuugaCcuuu...................	2	1	JCK-2
.................................................................................................................................................................................................................uuccacagcuuucuugaacuAu...................	3	1	JCK-1
.................................................................................................................................................................................................................uuccacagcuuucuugaacuuu...................	246	0	LCK-2
.................................................................................................................................................................................................................uuccacagcuuucuugaacuuu...................	250	0	LCK-1
.................................................................................................................................................................................................................uuccacagcuuucuugaaUuuu...................	13	1	LCK-2
.................................................................................................................................................................................................................uuccacagcuuucuugaacuuC...................	253	1	LCK-3
.................................................................................................................................................................................................................Cuccacagcuuucuugaacuuu...................	1	1	JT-1
.................................................................................................................................................................................................................uuccacagcuuucuugaacuuC...................	8	1	JT-2
.................................................................................................................................................................................................................Cuccacagcuuucuugaacuuu...................	1	1	JCK-1
.................................................................................................................................................................................................................uuccacagcuuucuugaacuuC...................	41	1	JT-1
.................................................................................................................................................................................................................uuccacagcuuucuugaaAuuu...................	4	1	JCK-1
.................................................................................................................................................................................................................uuccacagcuCucuugaacuuu...................	1	1	LT-1
.................................................................................................................................................................................................................uuccacagcuuuAuugaacuuu...................	4	1	LT-2
.................................................................................................................................................................................................................uuccacagcuuucuugaacuuA...................	50	1	JCK-1
.................................................................................................................................................................................................................uuccacagcuuucuugaacuuu...................	1108	0	JCK-3
.................................................................................................................................................................................................................uuccacagcuuucuugaaAuuu...................	3	1	JCK-2
.................................................................................................................................................................................................................uuccacagcuuucuugaacuuC...................	73	1	LT-1
.................................................................................................................................................................................................................uuccacagcuuCcuugaacuuu...................	1	1	LT-2
.................................................................................................................................................................................................................uuccacagcuuucuugaacAuu...................	4	1	JT-3
.................................................................................................................................................................................................................uNccacagcuuucuugaacuuu...................	2	1	LCK-3
.................................................................................................................................................................................................................uuccacagcuuuAuugaacuuu...................	2	1	JCK-1
.................................................................................................................................................................................................................uuccacagcuuGcuugaacuuu...................	2	1	JCK-1
.................................................................................................................................................................................................................uuccacagcuuucuugaacuuG...................	3	1	LT-3
.................................................................................................................................................................................................................uuccCcagcuuucuugaacuuu...................	1	1	LT-1
.................................................................................................................................................................................................................uuccCcagcuuucuugaacuuu...................	4	1	LT-3
.................................................................................................................................................................................................................uuccacagcuuucuugaacuuA...................	121	1	LT-3
.................................................................................................................................................................................................................uuccacagcuuucuugaacuuG...................	2	1	LT-1
.................................................................................................................................................................................................................uuccacagcuuucuugaacuuu...................	377	0	JT-1
.................................................................................................................................................................................................................uuNcacagcuuucuugaacuuu...................	4	1	LT-2
.................................................................................................................................................................................................................uuccacagcuuucuugaaUuuu...................	63	1	JCK-2
.................................................................................................................................................................................................................uuccaAagcuuucuugaacuuu...................	1	1	JCK-3
.................................................................................................................................................................................................................uuccacagcuuucNugaacuuu...................	1	1	LCK-1
.................................................................................................................................................................................................................uuccacagcCuucuugaacuuu...................	2	1	JCK-1
.................................................................................................................................................................................................................Cuccacagcuuucuugaacuuu...................	1	1	LT-3
.................................................................................................................................................................................................................uuUcacagcuuucuugaacuuu...................	1	1	LT-3
.................................................................................................................................................................................................................uuccacagcuuucuugaacuuu...................	2105	0	LCK-3
.................................................................................................................................................................................................................uuccacagcuuucuugaCcuuu...................	1	1	LCK-2
.................................................................................................................................................................................................................uuccacagcuuNcuugaacuuu...................	1	1	JT-1
.................................................................................................................................................................................................................uuccacagcCuucuugaacuuu...................	4	1	JCK-3
.................................................................................................................................................................................................................uuccacagcuuucuugCacuuu...................	1	1	JCK-2
.................................................................................................................................................................................................................uuccacagcuuucuugCacuuu...................	1	1	JT-3
.................................................................................................................................................................................................................uuccacagcuuucuugaacuuu...................	70	0	JT-2
.................................................................................................................................................................................................................uCccacagcuuucuugaacuuu...................	1	1	JCK-3
.................................................................................................................................................................................................................uuccacagcuuucuugaacuCu...................	1	1	LT-1
.................................................................................................................................................................................................................uuccaAagcuuucuugaacuuu...................	1	1	JCK-2
```

Star / Mature			
ucucuuucucucucucucucucuaaaaaaaacaaaaggaaagaaaaaaaaaaaaaaaaaacuuacuacuacuacuaguaguauaucuucuaauuugcuggaaaggcacaccaaagagaaagaaauuaaaccaaggaaggaaggaaggaaggaaggaagugagaucugaugagagaaaaagagaauguuuuugcaaguccuggucaugcuuuuccacagcuuucuugaacuuucuuucuuucuguuuagucu			
...uuccacagcuuucuugaacuuC...	89	1	JCK-2
...uuccacagcuuucuugaacCuu...	1	1	LCK-1
...uuccaNagcuuucuugaacuuu...	1	1	LCK-1
...uuccacagcuuucuugCacuuu...	2	1	LT-2
...uuccacagcuuucuugaacuAu...	16	1	JCK-2
...uuccacagcuuucuugaacuNu...	1	1	JT-2
...uuccacagcuGucuugaacuuu...	1	1	LT-2
...uuccaUagcuuucuugaacuuu...	1	1	LT-1
...uuccCcagcuuucuugaacuuu...	2	1	JCK-2
...uuccacagcuuucuugaacuCu...	1	1	LT-2
...uuccacagcuuucuugaacuAu...	17	1	LCK-3
...uuccacagcuuucuugaacGuu...	8	1	JCK-2
...uuccacagcuuucuugaaUuuu...	1	1	JT-3
...uucUacagcuuucuugaacuuu...	1	1	LCK-3
...uuccacagcuuucuugCacuuu...	2	1	LT-1
...uuccacagcuuucuugCacuuu...	1	1	JCK-1
...uuccacagcuuucuugaacuuu...	1512	0	LT-3
...uuccacagcuuucuugaacuuA...	31	1	LCK-2
...uuccacagcCuucuugaacuuu...	2	1	LT-2
...uuccacagcuuucuugaacGuu...	3	1	JCK-1
...uuccaAagcuuucuugaacuuu...	1	1	JCK-1
...uuccacagcuuucuugaacuCu...	2	1	JCK-1
...uuccacagcuuucuugaacuuA...	10	1	JT-2
...uuccacagcuuucuugaacuuuc...	16	0	JT-1
...uuccacagcuuucuugaacuuuc...	40	0	JCK-3
...uuccacagcuuucuugaacuuuG...	4	1	JCK-1
...uuccacagcuuucuugaacuuuA...	301	1	LCK-3
...uuccCcagcuuucuugaacuuuc...	1	1	LT-2
...uuccacagcuuucuugaacAuuc...	1	1	JCK-1
...uuccacagcuuucuugaacuuuG...	13	1	LT-1
...uuccacagcuuucuugaacuuCc...	1	1	LT-1
...uuccacagcuuucuugaacuuuA...	166	1	LT-3
...uuccacagcuuucuugaacuuuU...	9	1	JT-2
...uuccacagcuuucuugaacuuuG...	9	1	LCK-3
...uuccacagcuuucuugaacuuuU...	490	1	LCK-3
...uuccacagcuuucuugaacuuAc...	1	1	LT-2
...uuccacagcuuucuugaacuuuG...	11	1	LT-3
...uuccacagcuuucuugaacuuuG...	4	1	JCK-3
...uuccacagcuuucuugaacuuuA...	155	1	JCK-3
...uuccacagcuuucuugaacuAuc...	7	1	LT-2
...uuccacagcuuucuugaacuuuA...	230	1	LT-2
...uuccacagcuuucuugaacuuuA...	11	1	JT-2
...uuccacagcuuucuugaacuuuU...	306	1	LT-1
...uuccacagcuuucuugaacuuuc...	50	0	JCK-2
...uuccacagcuuucuugaacuuuU...	101	1	JT-1
...uuccacagcuuucuugaacuuuc...	3	0	JT-3
...uuccacCgcuuucuugaacuuuc...	1	1	LT-2
...uuccacagcuuucuugaacAuuc...	3	1	LT-3
...uuccacagcuuucuugaaUuuuc...	6	1	LCK-3
...uuccacagcuuucuugaacuuuc...	97	0	LCK-3
...uuccacGgcuuucuugaacuuuc...	1	1	LT-2
...uuccacagcuuucuugaacAuuc...	10	1	LCK-3
...uuccacagcuuucuugaacAuuc...	2	1	LT-2
...uuccacagcuuucuugaacuuuA...	40	1	LCK-1
...uuccacagcuuucuugaacuuuG...	9	1	LT-2
...uuccacagcuuucuugaacuuuc...	61	0	LT-2
...uuccacagcuuucuugaaUuuuc...	5	1	JCK-3
...uuccacagcuuucuugaacuuuA...	48	1	LCK-2
...uuccacagcuuucuugaacuuuA...	47	1	JT-1
...uuccacagcuuucuugaacuuuU...	140	1	LCK-1
...uuccacagcuuucuugaacuuuA...	16	1	JT-3
...uuccacagcuuucuugCacuuuc...	1	1	LT-2
...uuccacagcuuucuugaacAuuc...	1	1	JCK-3
...uuccacagcuuucuugaacuuuU...	369	1	JCK-3
...uuccacagcuuucuugaacuuAc...	1	1	LT-3
...uuccacagcuuucuugaaUuuuc...	3	1	JCK-2
...uuccacagcuuucuugaacuuuc...	28	0	LT-1
...uuNcacagcuuucuugaacuuuc...	1	1	LT-2
...uuccacagcuuucuugaacuuuU...	492	1	JCK-2
...uuccacagcuuucuugCacuuuc...	1	1	LCK-3

```
                                                                                                                                              Star                                                             Mature
ucucuuucucucucucucucucuaaaaaaacaaaaggaaagaaaaaaaaaaaaaaaaaacuuacuacuacuacuaguaguaucuucuaauuugcuggaaaggcacaccaaagagaaagaaauuaaaccaaggaaggaaggaaggaaggaaggaagugagaucugaugagagaaaaagagaauguuuuugcaaguccuggucaugcuuuuccacagcuuucuugaacuuucuuucuuucuguuuagucu
...............................................................................................................................................................................................................uuccacagcuuucuugaacuuuU..................   134   1   LCK-2
...............................................................................................................................................................................................................uuccacagcuuucuugaacuuAc..................   1     1   JT-2
...............................................................................................................................................................................................................uuccacagcuuucuugaacuAuc..................   4     1   LT-1
...............................................................................................................................................................................................................uuccacCgcuuucuugaacuuuc..................   1     1   LCK-3
...............................................................................................................................................................................................................uuccacagcuuucuugaaAuuuc..................   1     1   LT-3
...............................................................................................................................................................................................................uuccacagcuuucuugaacuuuU..................   18    1   JT-3
...............................................................................................................................................................................................................uuccacagcuuucuugaacuuuU..................   376   1   LT-2
...............................................................................................................................................................................................................uuccacagcuuucuugaacuuAc..................   5     1   JT-3
...............................................................................................................................................................................................................uuccacagcuuucuugaacuuuc..................   47    0   LT-3
...............................................................................................................................................................................................................uuccacagcuuucuugaacuuuU..................   324   1   LT-3
...............................................................................................................................................................................................................uuccacagcuuucuugaacuuuc..................   33    0   JCK-1
...............................................................................................................................................................................................................uuccacagcuuucuugaacuuuA..................   144   1   JCK-1
...............................................................................................................................................................................................................uuccacagcuuucuugaacuuuc..................   3     0   JT-2
...............................................................................................................................................................................................................uuccacagcuuucuugaacAuuc..................   4     1   LT-1
...............................................................................................................................................................................................................uuccacagcuuucuugaacuuuA..................   177   1   JCK-2
...............................................................................................................................................................................................................uuccacagcuuucuugaacuAuc..................   1     1   LT-3
...............................................................................................................................................................................................................uuccacagcuuucuugaacuuuU..................   294   1   JCK-1
...............................................................................................................................................................................................................uuccacagcuuucuugaacuuuG..................   8     1   JCK-2
...............................................................................................................................................................................................................uuccacagcuuucuugaaUuuuc..................   15    1   LCK-1
...............................................................................................................................................................................................................uuccacagcuuucuugaacuuuA..................   119   1   LT-1
...............................................................................................................................................................................................................uuccacagcuuucuugaaUuuuc..................   4     1   LT-1
...............................................................................................................................................................................................................uuccacagcuuucuugaaUuuuc..................   13    1   LCK-2
...............................................................................................................................................................................................................uuccacagcuuucuugaacuuAc..................   1     1   JCK-3
...............................................................................................................................................................................................................uuccacagcuuucuugaacuuCcu.................   1     1   LCK-3
...............................................................................................................................................................................................................uuccacagcuuucuugaacuuAcu.................   1     1   LT-1
...............................................................................................................................................................................................................uuccacagcuuucuugaacuuucA.................   4     1   JCK-3
...............................................................................................................................................................................................................uuccacagcuuucuugaacuuuUu.................   89    1   JCK-2
...............................................................................................................................................................................................................uuccacagcuuucuugaacuuuAu.................   5     1   LCK-3
...............................................................................................................................................................................................................uuccacagcuuucuugaacuuuUu.................   19    1   LT-1
...............................................................................................................................................................................................................uuccacagcuuucuugaacuuuUu.................   49    1   JCK-3
...............................................................................................................................................................................................................uuccacagcuuucuugaacuuAcu.................   2     1   JCK-2
...............................................................................................................................................................................................................uuccacagcuuucuugaacuuuAu.................   6     1   JCK-2
...............................................................................................................................................................................................................uuccacagcuuucuugaacuuucu.................   2     0   LCK-3
...............................................................................................................................................................................................................uuccacagcuuucuugaacuuAcu.................   2     1   LT-2
...............................................................................................................................................................................................................uuccacagcuuucuugaacuuucA.................   6     1   JT-1
...............................................................................................................................................................................................................uuccacagcuuucuugaacuuucA.................   2     1   JCK-2
...............................................................................................................................................................................................................uuccacagcuuucuugaacuuuAu.................   7     1   JCK-3
...............................................................................................................................................................................................................uuccacagcuuucuugaacuuucA.................   6     1   LT-2
...............................................................................................................................................................................................................uuccacagcuuucuugaacuuAcu.................   1     1   LT-3
...............................................................................................................................................................................................................uuccacagcuuucuugaacuAucu.................   1     1   JCK-1
...............................................................................................................................................................................................................uuccacagcuuucuugaacuuuUu.................   15    1   LT-2
...............................................................................................................................................................................................................uuccacagcuuucuugaacuAucu.................   1     1   JCK-3
...............................................................................................................................................................................................................uuccacagcuuucuugaacuAucu.................   4     1   JCK-2
...............................................................................................................................................................................................................uuccacagcuuucuugaacuuuAu.................   2     1   LT-2
...............................................................................................................................................................................................................uuccacagcuuucuugaacuuuUu.................   47    1   JCK-1
...............................................................................................................................................................................................................uuccacagcuuucuugaacuuucA.................   1     1   LT-1
...............................................................................................................................................................................................................uuccacagcuuucuugaacuuuAu.................   2     1   JCK-1
...............................................................................................................................................................................................................uuccacagcuuucuugaacuuucA.................   1     1   JCK-1
...............................................................................................................................................................................................................uuccacagcuuucuugaacuuucu.................   3     0   LT-1
...............................................................................................................................................................................................................uuccacagcuuucuugaacuAucu.................   5     1   LCK-3
...............................................................................................................................................................................................................uuccacagcuuucuugaacuuuAu.................   6     1   LT-3
...............................................................................................................................................................................................................uuccacagcuuucuugaacuuuUu.................   13    1   LCK-3
...............................................................................................................................................................................................................uuccacagcuuucuugaacuuuAu.................   2     1   LT-1
...............................................................................................................................................................................................................uuccacagcuuucuugaacuuuUu.................   13    1   LT-3
...............................................................................................................................................................................................................uuccacagcuuucuugaacuuuAu.................   4     1   JT-1
...............................................................................................................................................................................................................uuccacagcuuucuugaacuuucA.................   1     1   LT-3
...............................................................................................................................................................................................................uuccacagcuuucuugaacuuuAuu................   8     1   JCK-2
...............................................................................................................................................................................................................uuccacagcuuucuugaacuuuUuu................   9     1   JCK-1
...............................................................................................................................................................................................................uuccacagcuuucuugaacuuuUuu................   2     1   LCK-3
...............................................................................................................................................................................................................uuccacagcuuucuugaacuuuAuu................   7     1   LCK-3
...............................................................................................................................................................................................................uuccacagcuuucuugaacuuuUuu................   12    1   JCK-2
...............................................................................................................................................................................................................uuccacagcuuucuugaacuuuAuu................   3     1   LT-3
...............................................................................................................................................................................................................uuccacagcuuucuugaacuuuUuu................   1     1   LT-1
...............................................................................................................................................................................................................uuccacagcuuucuugaacuuuAuu................   5     1   LT-1
...............................................................................................................................................................................................................uuccacagcuuucuugaacuuucuA................   1     1   LT-2
...............................................................................................................................................................................................................uuccacagcuuucuugaacuuuAuu................   4     1   LT-2
...............................................................................................................................................................................................................uuccacagcuuucuugaacuuuGuu................   1     1   LT-3
...............................................................................................................................................................................................................uuccacagcuuucuugaacuuuAuu................   12    1   JCK-3
...............................................................................................................................................................................................................uuccacagcuuucuugaacuuuUuu................   5     1   LT-2
...............................................................................................................................................................................................................uuccacagcuuucuugaacuuAcuu................   3     1   JCK-2
```

```
                                                                                                                                                    Star                                            Mature
ucucuuucucucucucucucucucuaaaaaaaacaaaaggaaagaaaaaaaaaaaaaaaaaaacuuacuacuacuacuaguaguauaucuucuaauuugcuggaaaggcacaccaaagagaaagaaauuaaaccaaggaaggaaggaaggaaggaaggaagugagaucugaugagagaaaaagagaauguuuuugcaaguccuggucaugcuuuuccacagcuuucuugaacuuucuuucuuucuguuuagucu
.....................................................................................................................................................................................................................uuccacagcuuucuugaacuuuUuu................  2   1   LT-3
.....................................................................................................................................................................................................................uuccacagcuuucuugaacuuuUuu................  6   1   JCK-3
.....................................................................................................................................................................................................................uuccacagcuuucuugaacuuuAuu................  7   1   JCK-1
.....................................................................................................................................................................................................................uuccacagcuuucuugaacuuucuC................  1   1   LT-3
.....................................................................................................................................................................................................................uuccacagcuuucuugaacuuucuuu...............  1   0   JCK-1
.....................................................................................................................................................................................................................uuccacagcuuucuugaacuuuUuuu...............  2   1   LCK-3
.....................................................................................................................................................................................................................uuccacagcuuucuugaacuuuAuuu...............  5   1   JCK-3
.....................................................................................................................................................................................................................uuccacagcuuucuugaacuuuAuuu...............  3   1   JCK-1
.....................................................................................................................................................................................................................uuccacagcuuucuugaacuuuAuuu...............  3   1   LCK-3
.....................................................................................................................................................................................................................uuccacagcuuucuugaacuuuAuuu...............  3   1   JCK-2
......................................................................................................................................................................................................................uccacagcuuucuugaac......................  6   0   JT-1
......................................................................................................................................................................................................................uccacagcuuucuugaac......................  2   0   JT-2
......................................................................................................................................................................................................................uccacagcuuucuugaac......................  2   0   JCK-2
......................................................................................................................................................................................................................uccacagcuuucuugaac......................  2   0   JCK-3
......................................................................................................................................................................................................................uccacagcuuucuugaac......................  11  0   LT-3
......................................................................................................................................................................................................................uccacagcuuucuugaac......................  4   0   LT-1
......................................................................................................................................................................................................................uccacaUcuuucuugaac......................  1   1   LT-3
......................................................................................................................................................................................................................uccacagcuuucuugaac......................  4   0   JT-3
......................................................................................................................................................................................................................Cccacagcuuucuugaac......................  1   1   LT-1
......................................................................................................................................................................................................................uccacagcuCucuugaac......................  1   1   LT-3
......................................................................................................................................................................................................................ucNacagcuuucuugaac......................  1   1   LT-2
......................................................................................................................................................................................................................uccacagcuuucuugaac......................  10  0   LT-2
......................................................................................................................................................................................................................uccacagcuuucuugaac......................  13  0   LCK-3
......................................................................................................................................................................................................................uccacagcuuucuugaacu.....................  53  0   LCK-1
......................................................................................................................................................................................................................uccacagcuuucuugaacA.....................  1   1   LT-3
......................................................................................................................................................................................................................uccacagcuuucuugaacu.....................  12  0   LCK-3
......................................................................................................................................................................................................................uccacagcuuucuugaacu.....................  2   0   LT-2
......................................................................................................................................................................................................................uccacagcuuucuugaacu.....................  29  0   LCK-2
......................................................................................................................................................................................................................uccacagcuuucuugaacu.....................  7   0   LT-1
......................................................................................................................................................................................................................uccacagcuuucuugaaAu.....................  3   1   LT-2
......................................................................................................................................................................................................................uccacagcuuucuugaacu.....................  10  0   JCK-2
......................................................................................................................................................................................................................uccacagcuuucuugaacu.....................  2   0   JCK-1
......................................................................................................................................................................................................................uccacagcuuucuugaacu.....................  18  0   LT-3
......................................................................................................................................................................................................................uccacagcuuucuugaacu.....................  7   0   JCK-3
......................................................................................................................................................................................................................uccacagcuuucuugaaUu.....................  1   1   LT-1
......................................................................................................................................................................................................................uccacagcuuucuugaacA.....................  1   1   LT-1
......................................................................................................................................................................................................................uccacagcuuucuugaacuu....................  9   0   LCK-3
......................................................................................................................................................................................................................uccacagcuuucuugaacuu....................  5   0   JCK-3
......................................................................................................................................................................................................................uccacagcuuucuugaacuu....................  1   0   JCK-1
......................................................................................................................................................................................................................uccacagcuuucuugaacuu....................  2   0   LT-3
......................................................................................................................................................................................................................uccacagcuuucuugaaUuu....................  1   1   LT-3
......................................................................................................................................................................................................................uccacagcuuucuugaacuu....................  7   0   LT-2
......................................................................................................................................................................................................................uccacagcuuucuugaacuu....................  6   0   JCK-2
......................................................................................................................................................................................................................uccacagcuuucuugaacuu....................  2   0   LT-1
......................................................................................................................................................................................................................uccacagcuuucuugaacuuu...................  4   0   LT-3
......................................................................................................................................................................................................................uccacagcuuucuugaacuuu...................  2   0   JCK-1
......................................................................................................................................................................................................................uccacagcuuucuugaacuuC...................  1   1   LT-2
......................................................................................................................................................................................................................uccacagcuuucuugaacuuu...................  2   0   LT-1
......................................................................................................................................................................................................................Cccacagcuuucuugaacuuu...................  1   1   JCK-1
......................................................................................................................................................................................................................uccacagcuuucuugaacAuu...................  4   1   LT-2
......................................................................................................................................................................................................................uccacagcuuucuugaacuuu...................  5   0   LT-2
......................................................................................................................................................................................................................uccacagcuuucuugaacuuu...................  1   0   JCK-2
......................................................................................................................................................................................................................uccacagcuuucuugaacuuu...................  4   0   LCK-3
......................................................................................................................................................................................................................uccacagcuuucuugaacuuuc..................  2   0   LT-1
......................................................................................................................................................................................................................uccacagcuuucuugaacuuuA..................  10  1   LCK-3
......................................................................................................................................................................................................................uccacagcuuucuugaacuuuU..................  6   1   LCK-3
......................................................................................................................................................................................................................uccacagcuuucuugaacuuuU..................  1   1   LT-2
......................................................................................................................................................................................................................uccacagcuuucuugaacuuuc..................  1   0   JCK-2
......................................................................................................................................................................................................................uccacagcuuucuugaacuuuU..................  1   1   JCK-1
......................................................................................................................................................................................................................uccacagcuuucuugaacuuuU..................  3   1   JCK-2
......................................................................................................................................................................................................................uccacagcuuucuugaacuuuU..................  2   1   JCK-3
......................................................................................................................................................................................................................uccacagcuuucuugaacuuuU..................  2   1   LT-3
......................................................................................................................................................................................................................uccacagcuuucuugaacuuuc..................  1   0   JCK-1
......................................................................................................................................................................................................................uccacagcuuucuugaacuuuA..................  1   1   LT-2
......................................................................................................................................................................................................................uccacagcuuucuugaacuuuU..................  3   1   LT-1
......................................................................................................................................................................................................................uccacagcuuucuugaacuuuUu.................  2   1   LCK-3
.......................................................................................................................................................................................................................ccacagcuuucuugaacu.....................  36  0   LCK-2
.......................................................................................................................................................................................................................ccacagcuuucuugaacA.....................  2   1   LT-2
.......................................................................................................................................................................................................................ccacagcuuucuugaacu.....................  69  0   JCK-3
.......................................................................................................................................................................................................................ccacCgcuuucuugaacu.....................  1   1   LT-3
```

Star Mature

ucucuuucucucucucucucucuaaaaaaacaaaaggaaagaaaaaaaaaaaaaaaaacuuacuacuacuacuaguaguauaucuucuaauuugcuggaaaggcacaccaaagagaaagaaauuaaaccaaggaaggaaggaaggaaggaaggaagugagaucugaugagagaaaaagagaauguuuuugcaaguccuggucaugcuuuuccacagcuuucuugaacuuucuuucuuucuguuuagucu

ccacagcuuucuugaacu	318	0	LCK-3
ccacagcuuucuugaCcu	1	1	JT-1
ccacagcuuucuugaaUu	2	1	LT-1
ccacagcuuucuugaacu	342	0	LT-3
ccacagcuuucuugaacu	104	0	LT-1
ccacagcuuucuugaCcu	2	1	LCK-3
ccacagcuuucuugaCcu	1	1	LT-3
ccacagcuuucuugaacu	20	0	JT-3
ccacagcuuucuugaacu	14	0	JT-2
ccacagcuuuUuugaacu	4	1	LT-3
ccacagcuuucuugaacA	1	1	JCK-1
Acacagcuuucuugaacu	1	1	LT-2
ccacagcuuucuugaacu	89	0	JCK-2
ccacagcuuucuugCacu	2	1	LT-3
ccCcagcuuucuugaacu	1	1	LT-2
ccacagcuuucuugaacA	1	1	LT-3
ccacagcuuucuugaacu	301	0	LT-2
ccacagcuuucuugaacC	1	1	LT-3
ccacagcuuucuugaacu	51	0	JT-1
ccacagcuuucuugaaUu	2	1	LCK-3
ccacagcuuucuugaaUu	5	1	LT-3
ccacagcuuuAuugaacu	1	1	LT-3
ccacagcuuucuugaacu	51	0	JCK-1
ccacagcuuucuugaacG	1	1	LT-3
ccacagcuuucuugaacu	24	0	LCK-1
ccacagcCuucuugaacu	1	1	LT-3
ccacagcuuucuugaacA	1	1	JCK-2
ccacagcuuucuugaacC	2	1	JCK-1
ccacagcuuucuugaacuC	10	1	JT-1
ccacagcuuucuugaacuC	4	1	JCK-3
ccacagcuuucuugaacuC	3	1	LT-2
ccacagcCuucuugaacuu	3	1	LCK-3
ccacCgcuuucuugaacuu	1	1	JCK-2
ccacagcuuucuugaacuA	5	1	JT-3
ccacagcuuucuugaacuu	252	0	JCK-2
ccacagcuuucuugCacuu	2	1	JCK-3
cNacagcuuucuugaacuu	1	1	JCK-3
ccacagcuuucuugaCcuu	1	1	LT-2
ccacagcuuucuugaacuu	246	0	LT-2
ccCcagcuuucuugaacuu	1	1	LT-3
ccacCgcuuucuugaacuu	2	1	LCK-3
ccacagcuuucuugaacuC	5	1	LT-1
ccacagcuuucuugaacuu	168	0	JCK-1
ccaNagcuuucuugaacuu	1	1	JCK-1
ccacagcuuucuugaacuA	10	1	JT-1
ccacagcuuucuugCacuu	1	1	JCK-2
cNacagcuuucuugaacuu	1	1	JCK-2
ccacagcuuucuugaacuC	12	1	JCK-2
ccacagcuuucNugaacuu	1	1	JCK-1
ccacagcuuucuugaacuu	180	0	JCK-3
ccacagcuuucuugaacuu	34	0	JT-2
ccacagcuuucuugaacuA	3	1	JT-2
ccacagcuuucuugaCcuu	1	1	LCK-3
ccacagcuuucuugaacuA	8	1	JCK-3
ccacagcuuucuugaacuA	41	1	LCK-3
ccacagcuuucuugaacuu	42	0	JT-3
ccacagcuuucuugaaUuu	9	1	LT-2
ccacagcuuucuugaacuu	66	0	LT-1
ccacagcuuucuugaCcuu	1	1	JT-2
ccacagcuuucuugaaUuu	6	1	LCK-3
ccacagcuuucuugaacuC	2	1	JCK-1
ccacagcuuucuugaaUuu	4	1	JCK-3
cNacagcuuucuugaacuu	1	1	JT-1
ccacagcuuucuugaacuA	17	1	JCK-2
ccacagcuuucuugaacuA	9	1	LT-3
ccacagcuuucuugaaAuu	3	1	LT-3
ccacagcuuucuugaacuA	6	1	LT-1
ccacagcuuucuugaaAuu	1	1	JCK-2
ccacagcuuucuugaacuC	4	1	LT-3
ccacagcuuucuugaaUuu	3	1	JCK-1

Star Mature

ucucuuucucucucucucucuaaaaaaacaaaaggaaagaaaaaaaaaaaaaaaaaacuuacuacuacuacuaguaguauaucuucuaauuugcuggaaaggcacaccaaagagaaagaaauuaaaccaaggaaggaaggaaggaaggaaggaagugagaucugaugagagaaaaagagaauguuuuugcaaguccuggucaugcuuuuccacagcuuucuugaacuuucuuucuuucuguuuagucu

ccaAagcuuucuugaacuu	1	1	JCK-1
ccacagcuuucuugaacuu	68	0	JT-1
ccacagcuuucuugaacAu	1	1	JCK-1
ccacagcuuucuugaaUuu	3	1	LT-1
ccacagcuuucuugaacuA	11	1	JCK-1
ccacagcuuucuugaacuu	349	0	LCK-3
ccacagcuuucuugaaUuu	5	1	JCK-2
ccacagcuuucuugaacuu	251	0	LT-3
ccacagcuuucuugaacCu	1	1	LCK-3
ccacagcuuucuugaacuA	19	1	LT-2
ccacCgcuuucuugaacuu	1	1	JT-1
ccacagcuuucuugaaUuu	7	1	LT-3
ccacagcuuucuugCacuu	2	1	LT-2
ccacCgcuuucuugaacuuu	1	1	JT-1
ccacagcuuucuugaacuuC	18	1	JCK-3
ccacaAcuuucuugaacuuu	1	1	JCK-3
ccacagcuuucuugaacAuu	3	1	LT-2
ccacagcuuucuugaacuAu	6	1	JCK-1
ccacagcuuucuugaacuuA	53	1	JCK-3
ccacagGuuucuugaacuuu	1	1	JT-3
ccacagcuuucuugaacuCu	4	1	JCK-1
ccaAagcuuucuugaacuuu	1	1	LCK-1
ccacCgcuuucuugaacuuu	2	1	LT-2
ccacagcuuucuugaaUuuu	27	1	LCK-1
ccacagcuuucuugaacuuA	10	1	LT-1
ccacagcuuucuugaacuuG	1	1	JCK-3
ccacagcuuucuugaacuuA	48	1	LT-3
ccacagcuuucuugaacAuu	2	1	JCK-1
ccacagcuuucuugaacuuu	77	0	LCK-1
ccacagcuuucuugaacuuu	169	0	LT-1
ccacagcuuucuugaacuuu	21	0	JT-2
ccacagcuuucuugaacuuC	4	1	LT-1
ccacagcCuucuugaacuuu	1	1	JCK-3
ccacagcuuucuugaacuAu	9	1	LCK-3
ccacagcuuucuugaacAuu	2	1	LT-3
ccacagcuuucuugaacuuA	40	1	LCK-3
ccacagcuuucuugaacuCu	8	1	LT-3
ccacagcuuucuugaacuuu	31	0	JT-3
ccacagcuuucuugaacuuC	24	1	JCK-2
ccacagcuuucuugCacuuu	1	1	LCK-3
ccacagcuuucuugaacuAu	9	1	JCK-2
ccacagcuuucuugaacuuC	15	1	JCK-1
ccacagcuuucuugaacuuG	2	1	JCK-2
ccacagcuuucuugaacuCu	17	1	JCK-2
ccacagcuuucuugaacAuu	2	1	LT-1
ccacagcuuucuugaaUuuu	13	1	LCK-3
ccacagcuuucuugaacuuu	485	0	LT-2
ccacagcuuucuugaacuCu	4	1	LT-1
ccacCgcuuucuugaacuuu	3	1	JCK-3
ccacagcuuucuugaacAuu	1	1	JCK-2
ccacagcuuucuugaaUuuu	3	1	JCK-3
ccacagcuuucuugaacuuu	43	0	JT-1
ccacagcuuucuugaacuuA	41	1	JCK-1
ccacagcuuucuugaacuCu	7	1	JCK-3
ccacagcuuucuugaacAuu	5	1	LCK-3
ccacagcuuucuugaaUuuu	7	1	LT-3
ccacagcuuucuugaacuAu	5	1	JCK-3
ccacagcuuCcuugaacuuu	1	1	LT-3
ccacagcuuucuugCacuuu	1	1	JCK-2
ccacagcuuucuugaacuuA	11	1	JT-2
ccacagcuuucuugaacuAu	9	1	LT-3
ccacagcuuucuuAaacuuu	1	1	LT-2
ccacagcuuucuugaaUuuu	18	1	LCK-2
ccacagcuuucuugaacuCu	4	1	LT-2
ccacagcuuucuugaacuuC	5	1	LCK-3
ccacagcuuucuugCacuuu	1	1	LT-2
Ucacagcuuucuugaacuuu	1	1	JCK-1
ccacagcuuucuugaacuuu	439	0	LT-3
ccacagcuuucuugaacuuu	349	0	JCK-3
ccacCgcuuucuugaacuuu	1	1	JCK-1

Star Mature

ucucuuucucucucucucucucuaaaaaaacaaaaggaaagaaaaaaaaaaaaaaaaaacuuacuacuacuacuaguaguauaucuucuaauuugcuggaaaggcacaccaaagagaaagaaauuaaaccaaggaaggaaggaaggaaggaaggaagugagaucugaugagagaaaaagagaauguuuuugcaaguccuggucaugcuuuuccacagcuuucuugaacuuucuuucuuucuguuuagucu

ccacagcuuucuugaacuuC	20	1	LT-3
Ucacagcuuucuugaacuuu	4	1	JCK-2
ccacagcuuucuugaacuuC	14	1	LT-2
ccacaNcuuucuugaacuuu	1	1	LCK-1
ccacagcuuucuugaCcuuu	1	1	JCK-3
ccacagcuuucuugNacuuu	1	1	LCK-1
ccacagcuuucuugaacGuu	1	1	JCK-1
ccacagcuuucuugaacuuA	69	1	JCK-2
ccacagcuuucuugaacuuu	506	0	LCK-3
ccacagcuuucuugaGcuuu	1	1	LCK-2
ccacagcuuucuugaacuuu	495	0	JCK-2
ccacagcuuucuugaacuuG	1	1	LT-1
ccacagcuuucuugaacuuG	7	1	JCK-1
cNacagcuuucuugaacuuu	1	1	JCK-1
ccacagcuuucuugaaAuuu	5	1	LCK-3
ccacagcuuucuugaacuuu	71	0	LCK-2
ccacagcuuucuugaacuuA	8	1	JT-3
ccacagcuuucuugaacuuA	51	1	LT-2
ccacagcuuucuugaacuAu	16	1	LT-2
ccacagcuuucuugaaUuuu	4	1	JCK-1
ccacagcuuucuugaacuuG	1	1	LT-3
ccacagcuuucuugaacuuu	334	0	JCK-1
ccNcagcuuucuugaacuuu	1	1	LCK-1
ccacagcuuucuugaacuuuU	349	1	LCK-3
ccacagcuuucuugaacuuuU	223	1	LT-3
ccacagcuuucuugaacuuuc	8	0	JCK-2
ccacagcuuucuugaacuuAc	5	1	LCK-3
ccacagcuuucuugaacuuuU	138	1	JCK-1
ccacagcuuucuugaacuuAc	3	1	LT-3
ccacagcuuucuugaacuuuA	109	1	LCK-3
ccacagcuuucuugaacuuuA	7	1	JT-1
ccacagcuuucuugaacuuuU	153	1	JCK-3
ccacagcuuucuugaacuuCc	1	1	LT-3
ccacagcuuucuugaacuuuU	2	1	JT-2
ccacagcuuucuugaacuuuG	8	1	JCK-2
ccacagcuuucuugaacuuCc	1	1	JCK-1
ccacagcuuucuugaacuuuA	178	1	JCK-2
ccacagcuuucuugaacuAuc	7	1	LCK-3
ccacagcuuucuugaacuuuG	6	1	LT-1
ccacagcuuucuugaacuuuc	9	0	JCK-3
ccacagcuuucuugaacuuuU	42	1	JT-1
ccacagcuuucuugaacuuCc	1	1	LCK-3
ccacagcuuucuugaacuuuG	10	1	JCK-3
ccacagcuuucuugaacuuuc	16	0	LT-2
ccacagcuuucuugaacuuuU	52	1	LCK-1
ccacagcuuucuugaacuuAc	1	1	LT-2
ccacagcuuucuugaacuuuA	82	1	LT-3
ccacagcuuucuugaacuuuG	9	1	JCK-1
ccacagcuuucuugaacuuuG	14	1	LT-2
ccacagcuuucuugaacuAuc	1	1	LT-3
ccacagcuuucuugaacuuuA	33	1	LT-1
ccacagcuuucuugaacuuuc	30	0	LCK-2
ccacagcuuucuugaacuuuc	4	0	JCK-1
ccacagcuuucuugaacuuuU	87	1	LT-1
ccacagcuuucuugaacuuuG	6	1	LT-3
ccacagcuuucuugaacuuuU	13	1	JT-3
ccacagcuuucuugaacuuAc	1	1	JCK-2
ccacagcuuucuugaacuuuA	90	1	JCK-3
ccacagcuuucuugaacuuuU	64	1	LCK-2
ccacagcuuucuugaacuuuc	27	0	LCK-1
ccacagcuuucuugaacuuuc	8	0	LT-3
ccacagcuuucuugaacuuuU	246	1	LT-2
ccacagcuuucuugaaUuuuc	1	1	LT-2
ccacagcuuucuugaacuuuA	150	1	JCK-1
ccacagcuuucuugaacuuuc	3	0	LT-1
ccacagcuuucuugaacuuuU	184	1	JCK-2
ccacagcuuucuugaacuuuA	146	1	LT-2
ccacagcuuucuugaacuuuc	29	0	LCK-3
ccacagcuuucuugaCcuuuc	1	1	LT-1
ccacagcuuucuugaacuuuG	17	1	LCK-3

Star Mature

ucucuuucucucucucucucucucuaaaaaaaacaaaaggaaagaaaaaaaaaaaaaaaaacuuacuacuacuacuaguaguauaucuucuaauuugcuggaaaggcacaccaaagagaaagaaauuaaaccaaggaaggaaggaaggaaggaaggaagugagaucugaugagagaaaaagagaauguuuuugcaaguccuggucaugcuuuuccacagcuuucuugaacuuucuuucuuucuguuuagucu

ccacagcuuucuugaacuuuUu	7	1	LT-1
ccacagcuuucuugaacuuuAu	16	1	JCK-3
ccacagcuuucuugaacuuAcu	23	1	LCK-3
ccacagcuuucuugaacuuAcu	1	1	JCK-1
ccacagcuuucuugaacuuuAu	1	1	JT-3
ccacagcuuucuugaacuuuAu	19	1	JCK-1
ccacagcuuucuugaacuuuUu	29	1	JCK-1
ccacagcuuucuugaacuuucu	1	0	JCK-1
ccacagcuuucuugaacuuuAu	23	1	LCK-3
ccacagcuuucuugaacuuuAu	16	1	LT-2
ccacagcuuucuugaacuuAcu	1	1	LT-1
ccacagcuuucuugaacuuucG	1	1	JCK-3
ccacagcuuucuugaacuuuGu	3	1	LT-1
ccacagcuuucuugaacuuucA	3	1	JCK-3
ccacagcuuucuugaacuuucu	4	0	JCK-2
ccacagcuuucuugaacuuucu	1	0	LT-1
ccacagcuuucuugaacuuuUu	39	1	LT-2
ccacagcuuucuugaacuuucA	3	1	LT-3
ccacagcuuucuugaacuuuUu	38	1	JCK-3
ccacagcuuucuugaacuuuUu	22	1	LCK-3
ccacagcuuucuugaacuAucu	2	1	LCK-3
ccacagcuuucuugaacuuuUu	54	1	JCK-2
ccacagcuuucuugaacuuucA	5	1	JCK-2
ccacagcuuucuugaacuuuAu	4	1	LT-1
ccacagcuuucuugaacuuAcu	2	1	LT-3
ccacagcuuucuugaacuuucA	1	1	JCK-1
ccacagcuuucuugaacuuucA	4	1	LT-2
ccacagcuuucuugaacuuuUu	4	1	JT-3
ccacagcuuucuugaacuuucu	3	0	LT-3
ccacagcuuucuugaacuuucu	4	0	JCK-3
ccacagcuuucuugaacuuuUu	25	1	LT-3
ccacagcuuucuugaacuuuAu	23	1	JCK-2
ccacagcuuucuugaacuuuAu	13	1	LT-3
ccacagcuuucuugaacuuAcu	4	1	JCK-2
ccacagcuuucuugaacuuucC	2	1	LT-3
ccacagcuuucuugaacuuAcu	4	1	JCK-3
ccacagcuuucuugaacuuucG	5	1	JCK-2
ccacagcuuucuugaacuuuUu	2	1	JT-2
ccacagcuuucuugaacuuuGu	5	1	LCK-3
ccacagcuuucuugaacuuAcu	3	1	LT-2
ccacagcuuucuugaacuuuAuu	4	1	LT-3
ccacagcuuucuugaacuuuUuu	14	1	LCK-3
ccacagcuuucuugaacuuuAuu	4	1	JT-3
ccacagcuuucuugaacuuuGuu	1	1	JCK-2
ccacagcuuucuugaacuuucCu	1	1	JCK-2
ccacagcuuucuugaacuuuUuu	4	1	LT-3
ccacagcuuucuugaacuuuUuu	11	1	JCK-2
ccacagcuuucuugaacuuucAu	1	1	JCK-2
ccacagcuuucuugaacuuuAuu	1	1	JT-2
ccacagcuuucuugaacuuuAuu	21	1	JCK-2
ccacagcuuucuugaacuuuAuu	13	1	JCK-3
ccacagcuuucuugaacuuuUuu	4	1	JCK-1
ccacagcuuucuugaacuuucuA	2	1	LCK-3
ccacagcuuucuugaacuuuUuu	9	1	JCK-3
ccacagcuuucuugaacuuuAuu	14	1	JCK-1
ccacagcuuucuugaacuuuAuu	25	1	LCK-3
ccacagcuuucuugaacuuuUuu	5	1	LT-1
ccacagcuuucuugaacuuucuu	1	0	JCK-3
ccacagcuuucuugaacuuucAu	1	1	JCK-3
ccacagcuuucuugaacuuuAuu	3	1	LT-1
ccacagcuuucuugaacuuucuA	1	1	LT-2
ccacagcuuucuugaacuuuAuu	13	1	LT-2
ccacagcuuucuugaacuuuUuu	5	1	LT-2
ccacagcuuucuugaacuuuAuuu	1	1	LT-2
ccacagcuuucuugaacuuuAuuu	1	1	LT-1
ccacagcuuucuugaacuuuUuuu	2	1	JCK-2
ccacagcuuucuugaacuuuUuuu	3	1	LT-3
ccacagcuuucuugaacuuuAuuu	1	1	JCK-3
ccacagcuuucuugaacuuuAuuu	1	1	LT-3
ccacagcuuucuugaacuuuUuuu	2	1	JCK-1

```
                                                                                                                                                                      Star                                                                   Mature
ucucuuucucucucucucucucuaaaaaaaacaaaaggaaagaaaaaaaaaaaaaaaaaaacuuacuacuacuacuaguaguauaucuucuaauuugcuggaaaggcacaccaaagagaaagaaauuaaaccaaggaaggaaggaaggaaggaaggaagugagaucugaugagagaaaaagagaauguuuuugcaaguccuggucaugcuuuuccacagcuuucuugaacuuucuuucuuucuguuuagucu
.....................................................................................................................................................................................................................ccacagcuuucuugaacuuuUuuu...............   3   1   JCK-3
.....................................................................................................................................................................................................................ccacagcuuucuugaacuuucuCu...............   1   1   JCK-2
.....................................................................................................................................................................................................................ccacagcuuucuugaacuuuAuuu...............   3   1   JCK-1
.....................................................................................................................................................................................................................ccacagcuuucuugaacuuucAuu...............   1   1   JCK-2
.....................................................................................................................................................................................................................ccacagcuuucuugaacuuuUuuu...............   1   1   LT-2
.....................................................................................................................................................................................................................ccacagcuuucuugaacuuuAuuu...............   2   1   JCK-2
......................................................................................................................................................................................................................cCcagcuuucuugaacuu....................   1   1   LCK-3
......................................................................................................................................................................................................................cacagcuuucuugaacuu....................   1   0   LT-3
......................................................................................................................................................................................................................cacagcuuucuugaacuu....................   3   0   JCK-3
......................................................................................................................................................................................................................cacagcuuucuugaacuu....................   3   0   LT-2
......................................................................................................................................................................................................................cacagcuuucuugaacuu....................   5   0   LCK-3
......................................................................................................................................................................................................................cacagcuuucuugaacuuA...................   1   1   JCK-1
......................................................................................................................................................................................................................cacagcuuucuugaacuuu...................   2   0   JCK-3
......................................................................................................................................................................................................................cacagcuuucuugaacuuA...................   2   1   JCK-3
......................................................................................................................................................................................................................cacagcuuucuugaacuuu...................   10  0   JT-3
......................................................................................................................................................................................................................cacagcuuucuugaacuuA...................   1   1   LT-3
......................................................................................................................................................................................................................cacagcuuucuugaacuuu...................   4   0   LT-2
......................................................................................................................................................................................................................cacagcuuucuugaacuuu...................   6   0   LT-3
......................................................................................................................................................................................................................cacagcuuucuugaacuuu...................   1   0   JCK-1
......................................................................................................................................................................................................................cacagcuuucuugaacuAu...................   2   1   LT-2
......................................................................................................................................................................................................................cacagcuuucuugaacuuu...................   1   0   JT-2
......................................................................................................................................................................................................................cacagcuuucuugaacuuu...................   8   0   JCK-2
......................................................................................................................................................................................................................cacagcuuucuugaacuuu...................   2   0   LT-1
......................................................................................................................................................................................................................cacagcuuucuugaacuuuA..................   1   1   JCK-3
......................................................................................................................................................................................................................cacagcuuucuugaacuuuU..................   1   1   LT-1
......................................................................................................................................................................................................................cacagcuuucuugaacuuuU..................   5   1   JCK-3
......................................................................................................................................................................................................................cacagcuuucuugaacuuuA..................   4   1   LCK-3
......................................................................................................................................................................................................................cacagcuuucuugaacuuuU..................   2   1   LT-2
......................................................................................................................................................................................................................cacagcuuucuugaacuuuU..................   5   1   JCK-2
......................................................................................................................................................................................................................cacagcuuucuugaacuuuU..................   5   1   LCK-3
......................................................................................................................................................................................................................cacagcuuucuugaacuuuU..................   2   1   LT-3
......................................................................................................................................................................................................................cacagcuuucuugaacuuuU..................   3   1   JCK-1
......................................................................................................................................................................................................................cacagcuuucuugaacuuuA..................   1   1   LT-3
......................................................................................................................................................................................................................cacagcuuucuugaacuuucu.................   2   0   JCK-2
......................................................................................................................................................................................................................cacagcuuucuugaacuuucu.................   3   0   LT-1
......................................................................................................................................................................................................................cacagcuuucuugaacuuucu.................   2   0   JCK-1
......................................................................................................................................................................................................................cacagcuuucuugaacuuucu.................   9   0   JCK-3
......................................................................................................................................................................................................................cacagcuuucuugaacuuuAuu................   2   1   JCK-3
......................................................................................................................................................................................................................cacagcuuucuugaacuuuUuu................   2   1   LT-3
......................................................................................................................................................................................................................cacagcuuucuugaacuuuAuu................   3   1   JCK-1
......................................................................................................................................................................................................................cacagcuuucuugaacuuuUuu................   1   1   LT-1
......................................................................................................................................................................................................................cacagcuuucuugaacuuuAuu................   8   1   JCK-2
......................................................................................................................................................................................................................cacagcuuucuugaacuuAcuuu...............   1   1   LT-1
.......................................................................................................................................................................................................................acagcuuucuugaacuuu...................   1   0   LT-2
.......................................................................................................................................................................................................................acagcuuucuugaacuuA...................   2   1   LT-2
.......................................................................................................................................................................................................................acagcuuucuugaaUuuu...................   6   1   LCK-3
.......................................................................................................................................................................................................................acagcuuucuugaacuuuU..................   1   1   LT-3
.......................................................................................................................................................................................................................acagcuuucuugaacuuuA..................   1   1   LT-1
.......................................................................................................................................................................................................................acagcuuucuugaacuuuU..................   3   1   LT-2
.......................................................................................................................................................................................................................acagcuuucuugaacuuuU..................   4   1   LCK-3
.......................................................................................................................................................................................................................acagcuuucuugaacuuuUu.................   3   1   LT-3
......................................................................................................................................................................................................................................uuucuGucuuucuguuua....   1   1   LT-2
```
